# CircPIAS1 promotes hepatocellular carcinoma progression by inhibiting ferroptosis via the miR-455-3p/NUPR1/FTH1 axis

**DOI:** 10.1186/s12943-024-02030-x

**Published:** 2024-05-28

**Authors:** Xiao-Yu Zhang, Shan-Shan Li, Yu-Rong Gu, Le-Xin Xiao, Xin-Yi Ma, Xin-Ru Chen, Jia-Liang Wang, Chun-Hong Liao, Bing-Liang Lin, Yue-Hua Huang, Yi-Fan Lian

**Affiliations:** 1 Guangdong Provincial Key Laboratory of Liver Disease Research, The Third Affiliated Hospital of Sun Yat-sen University, Guangzhou, China; 2 Department of Infectious Diseases, The Third Affiliated Hospital of Sun Yat-sen University, Guangzhou, China; 3 Department of Medical Oncology, The Sixth Affiliated Hospital of Sun Yat-Sen University, Guangzhou, China; 4 Key Laboratory of Tropical Disease Control, Sun Yat-sen University, Ministry of Education, Guangzhou, China

**Keywords:** circPIAS1, Hepatocellular carcinoma, miR-455-3p, NUPR1, Ferroptosis

## Abstract

**Background:**

The role of circRNAs in hepatocellular carcinoma (HCC) progression remains unclear. CircPIAS1 (circBase ID: hsa_circ_0007088) was identified as overexpressed in HCC cases through bioinformatics analysis. This study aimed to investigate the oncogenic properties and mechanisms of circPIAS1 in HCC development.

**Methods:**

Functional analyses were conducted to assess circPIAS1’s impact on HCC cell proliferation, migration, and ferroptosis. Xenograft mouse models were employed to evaluate circPIAS1’s effects on tumor growth and pulmonary metastasis in vivo. Bioinformatics analysis, RNA immunoprecipitation, and luciferase reporter assays were utilized to elucidate the molecular pathways influenced by circPIAS1. Additional techniques, including RNA pulldown, fluorescence in situ hybridization (FISH), chromatin immunoprecipitation (ChIP), qPCR, and western blotting, were used to further explore the underlying mechanisms.

**Results:**

CircPIAS1 expression was elevated in HCC tissues and cells. Silencing circPIAS1 suppressed HCC cell proliferation and migration both in vitro and in vivo. Mechanically, circPIAS1 overexpression inhibited ferroptosis by competitively binding to miR-455-3p, leading to upregulation of Nuclear Protein 1 (NUPR1). Furthermore, NUPR1 promoted FTH1 transcription, enhancing iron storage in HCC cells and conferring resistance to ferroptosis. Treatment with ZZW-115, an NUPR1 inhibitor, reversed the tumor-promoting effects of circPIAS1 and sensitized HCC cells to lenvatinib.

**Conclusion:**

This study highlights the critical role of circPIAS1 in HCC progression through modulation of ferroptosis. Targeting the circPIAS1/miR-455-3p/NUPR1/FTH1 regulatory axis may represent a promising therapeutic strategy for HCC.

**Supplementary Information:**

The online version contains supplementary material available at 10.1186/s12943-024-02030-x.

## Introduction

Hepatocellular carcinoma (HCC) is the predominant form of liver cancer globally, representing 75–85% of cases and ranking as a leading cause of cancer-related mortality [1]. Despite advancements in treatment modalities such as ablation, locoregional interventions, resection, and targeted drug therapies tailored to tumor characteristics and disease progression, the 5-year survival rates for HCC patients remain suboptimal due to challenges posed by metastasis and drug resistance [2, 3]. The molecular underpinnings governing HCC pathogenesis, metastasis, and treatment resistance are not fully elucidated, highlighting the critical need for identifying novel biomarkers, elucidating their molecular functions, and developing targeted therapeutic strategies.

Circular RNAs (circRNAs) have emerged as pivotal players in cancer biology. These unique endogenous non-coding RNAs are formed through the back-splicing of intronic or exonic sequences, resulting in a covalently closed-loop structure that confers enhanced stability by lacking 3′ and 5′ ends [4]. CircRNAs function as sponges for microRNAs (miRNAs) or binders of proteins, thereby modulating gene expression [5]. Alterations in circRNA expression levels have been observed in various cancers, underscoring their significance in tumor biology [6–8]. For instance, circMDK has been shown to promote HCC tumorigenesis and progression by upregulating ATG16L1 expression through the sequestration of miR-346 and miR-874-3p [9], whereas circGPR137B inhibits HCC growth and metastasis via the circGPR137B/miR-4739/FTO feedback loop, presenting a potential therapeutic target in HCC treatment [10]. Additionally, Wang et al. demonstrated that circRAPGEF5 collaborates with RBFOX2 to confer resistance to ferroptosis in endometrial cancer by modulating the alternative splicing of TFRC [11]. However, the possibility that HCC-related circRNAs exert tumorigenic effects through other regulatory mechanisms warrants further investigation.

Ferroptosis is a form of cell death driven by iron and lipid metabolism, closely associated with oxidative stress and inflammation, and distinct from autophagy, apoptosis, and necrosis [12]. Characterized by a decline in glutathione (GSH), iron-induced lipid peroxidation, and mitochondrial changes, ferroptosis is considered a promising target for cancer therapy [13, 14]. Studies have shown that ferroptosis agonists can overcome drug resistance and inhibit tumor metastasis, with enhanced anti-tumor effects observed when combined with other treatments [15]. Nuclear Protein 1 (NUPR1) is a key regulatory molecule involved in various cellular processes, including gene expression, cell cycle progression, apoptosis, and stress responses [16]. NUPR1 plays a critical role in the interplay between oxidative stress and ferroptosis [17]. Several regulatory pathways indicate NUPR1’s involvement in ferroptosis. For example, RNF113A knockout significantly reduces cisplatin-induced NUPR1 overexpression, leading to increased lipid peroxidation [18]. The NUPR1 inhibitor ZZW-115 downregulates TFAM, affecting the antioxidant system and promoting ferroptosis in pancreatic cancer cells [17]. Additionally, Liu et al. demonstrated that NUPR1 transcriptionally activates LCN2, regulating iron metabolism, inhibiting ferroptosis in cancer cells, and promoting pancreatic cancer progression [19]. Therefore, NUPR1 acts as a key regulator of ferroptosis, contributing to ferroptosis resistance in cancer cells by modulating iron metabolism and enhancing antioxidant-related gene expression.

In our study, we identified a novel circular RNA, circPIAS1 (circBase ID: hsa_circ_0007088), which is significantly elevated in HCC patients and closely associated with patient outcomes. Our findings elucidate the role of circPIAS1 in HCC development and its underlying mechanism, offering potential therapeutic targets for HCC treatment.

## Materials and methods

### Bioinformatics analysis

The online tools ENCORI (http://starbase.sysu.edu.cn/index.php), CircInteractome(https://circinteractome.nia.nih.gov/) and miRDB (http://www.mirdb.org/) were used for predicting potential interactions between circRNAs and miRNAs [20, 21]. miRDB, ENCORI, and TargetScan (http://www.targetscan.org/mamm_31/) were utilized to predict miRNA target genes. HCC cohort datasets from The Cancer Genome Atlas (TCGA) (https://www.cancer.gov/ccg/research/genome-sequencing/tcga), Gene Expression Profiling Interactive Analysis (GEPIA) (http://gepia.cancer-pku.cn/), and Gene Expression Omnibus (GEO) (https://www.ncbi.nlm.nih.gov/geo/) were analyzed to compare gene expression levels between tumor and normal tissues, and to assess gene correlations. Gene Set Enrichment Analysis (GSEA) was conducted using the R package “Cluster Profile” to investigate biological functions in control and sh-circPIAS1 HCC cells.

### Reagents

The following commercially available antibodies were utilized: NUPR1 (15056-1-AP, Proteintech, 1:1000 for WB, and 1:400 for IHC), GAPDH (60004-1-Ig, Proteintech, 1:2000 for WB), FTH1 (4393T, Cell Signaling Technology, 1:1000 for WB and 1:400 for IHC), GPX4 (52455T, Cell Signaling Technology, 1:1000 for WB), SLC11A2 (15083T, Cell Signaling Technology, 1:1000 for WB), SLC7A11 (12691T, Cell Signaling Technology, 1:1000 for WB), KEAP1 (8047T, Cell Signaling Technology, 1:1000 for WB), NRF2 (12721T, Cell Signaling Technology, 1:1000 for WB), Ago2 (07-590, Merck Millipore), IgG rabbit (7074 S, Cell Signaling Technology), IgG mouse (12-371B, Merck Millipore). Small molecule inhibitors included ZZW-115 (HY-111,838, MedchemExpress), Z-VAD-FMK (S7023, Selleck), Necrostatin-1 (S8037, Selleck), Ferrostatin-1 (S7243, Selleck), and Liproxstatin-1 (S7699, Selleck). All other chemical reagents were obtained from Sigma-Aldrich, unless specified otherwise.

### Tissue samples

Thirty-six paraffin-embedded HCC samples were obtained from Sun Yat-sen University Cancer Center (Guangzhou, China) survival prognosis analysis. To compare circPIAS1 levels in HCC versus non-tumor tissues, 8 pairs of fresh HCC samples and their adjacent non-cancerous counterparts were obtained from the Third Affiliated Hospital of Sun Yat-sen University. A surgical tumor resection was performed on each patient in the department of hepatobiliary surgery. The study was approved by the Institute Research Ethics Committee at the Sun Yat-sen University Cancer Center and the Third Affiliated Hospital of Sun Yat-sen University. Written informed consent was obtained from each patient.

### Immunohistochemistry (IHC) analysis

Tissue samples were paraffin-embedded and cut into 5-µm sections. The slides were then heated at 65 °C for 120 min, followed by de-paraffinized, hydrated and antigen retrieval. Endogenous peroxidases were blocked with 3% peroxide for 15 min, then in 5% BSA for 1 h and incubated overnight at 4 °C with the indicated primary antibodies. Subsequently, anti-rabbit/mouse secondary antibodies (K5007, Dako) were applied and incubated for 60 min at 37 °C. Signals were revealed using freshly prepared DAB substrate solution (K5007, Dako) at room temperature for 5 min. Finally, the sections were counterstained with Mayer’s hematoxylin, dehydrated, and mounted. Staining results were captured using microscopy (DM4000B, Leica). Each section was evaluated by two independent pathologists blinded to the clinical status of patients and graded according to the positive staining intensity scores (no staining, 0; weak staining, 1; moderate staining, 2; strong staining, 3) and the expression extent scores (< 25%, 1; 25–50%, 2; 50–75%, 3; > 75%, 4).

### CircPIAS1 RNAScope in situ hybridization assay

CircPIAS1 expression in HCC tissues was examined using RNA in situ hybridization with the BaseScope™ RED Kit 2 (Advanced Cell Diagnostics, USA), following the manufacturer’s protocol. A custom-designed probe specific to circPIAS1 was employed. Detection sensitivity for CircPIAS1 molecules was achieved at the single-copy level. Quantification of single-molecule signals was conducted on a cell-by-cell basis through manual counting. A probe targeting human PPIB mRNA (476,701, Advanced Cell Diagnostics) was used as the positive control, while a probe targeting Bacillus subtilis DapB mRNA (310,043, Advanced Cell Diagnostics) served as the negative control. Cells were considered positive if they displayed a visible red dot or cluster at 40× magnification under a microscope (DM4000B, Leica). Scoring criteria were as follows: circPIAS1 low, 0 ≤ positive cells < 3 per 20 cells; circPIAS1 high, positive cells ≥ 3 per 20 cells.

### Cell culture

The human embryonic kidney cell line HEK-293T, several human HCC cell lines (Huh7, PLC/PRF/5, Hep3B, LM3, MHCC-97 H, MHCC-97 L, SNU449, SNU387, and HepG2), and two immortalized hepatic cell lines (MIHA and LO2) were sourced from the College of Life Sciences, Sun Yat-sen University and employed in this study. All cell lines were cultured in Dulbecco’s Modified Eagle’s Medium (DMEM, Invitrogen) supplemented with 10% fetal bovine serum (FBS, Gibco) at 37 °C and 5% CO_2_. Thawed from early passage stocks, the cells were regularly subcultured every 2 days. Bimonthly PCR assays were conducted to confirm the absence of mycoplasma contamination.

### Reverse transcriptase PCR (RT-PCR) and quantitative real-time PCR (qPCR)

RNA was extracted using TRIzol (T9424, Sigma-Aldrich) and reverse transcribed with the GoScript system (A5001, Promega). RT-PCR was performed using PrimeSTAR Master Mix (R045A, Takara) according to the manufacturer’s instructions, including PCR control. Products were separated on a 2% agarose gel and visualized with GelRed (D0140, Beyotime). qPCR was conducted on a LightCycler 480 (Roche) with Platinum SYBR Green mix (11,744,500, Invitrogen). CircRNA, mRNA, and miRNA expression levels were normalized to GAPDH and U6, respectively, using the 2^−ΔΔCt^ method for determining gene expression level [20]. Primer sequences are listed in Supplementary Table 1.

### Plasmid construction, oligonucleotide synthesis and transfection

The pLO5-ciR plasmid (Geneseed, Guangzhou, China), containing the sequence of circPIAS1, was constructed and used to upregulate circPIAS1 expression. Two specific short hairpin RNAs (shRNAs) targeting the covalent closed junction of circPIAS1 were cloned into the pLKO.1 plasmid (Sigma-Aldrich). The coding sequence of NUPR1 with a C-terminus Flag tag was constructed in the pcDNA3.1(+) plasmid (Invitrogen). Correct constructs were confirmed by DNA sequencing. The oligonucleotides of the miR-455-3p mimic/inhibitor and controls were synthesized by RiboBio (Guangzhou, China). Plasmids and oligonucleotides were transfected using ViaFect Transfection Reagent (Promega) or Lipofectamine RNAiMax (Invitrogen), respectively, according to the manufacturer’s instructions. The shRNA sequences targeting the circPIAS1 covalent closed junction were as follows: sh-circPIAS1#1, 5’-TATTGATGGCATCAGACAACA-3’, and sh-circPIAS1#2, 5’-TTGATGGCATCAGACAACAGT-3’. The non-targeting shRNA sequence served as a negative control: shNC, 5’-CAACAAGATGAAGAGCACCAA-3’.

### Lentivirus packaging and infection

Lentiviruses for circPIAS1 overexpression and knockdown were produced by co-transfecting constructed plasmids and the packaging plasmids psPAX2 and pMD2.G (Addgene) into 293T cells for 48 h. Culture supernatants containing lentivirus were collected, filtered, and concentrated. Lentiviruses expressing miR-455-3p (pHBLV-h-miR-455-3p-puro) or miR-455-3p inhibitor (pHBLV-h-sh-miR-455-3p-puro) were purchased from HanBio (Shanghai, China). HCC cells were infected with lentivirus in the presence of 8 µg/mL polybrene (Sigma-Aldrich). Infected cells were screened with 2 µg/mL puromycin (Merck) for 2 weeks, and successful establishment was confirmed by qPCR.

### Luciferase reporter assay

Reporter plasmids containing the wild-type or mutant miR-455-3p putative binding site were constructed by inserting the circPIAS1 and 3’UTR of NUPR1 sequences into the pMIR-REPORT vector (Invitrogen). These constructs were then co-transfected with miR-455-3p mimics or inhibitors. The FTH1 promoter sequence was inserted into the pGL3-reporter vector (Promega). Luciferase activities of all reporter vectors were evaluated using the dual luciferase reporter assay system (Promega), with Renilla luciferase used for normalization.

### RNase R and actinomycin D treatment

For RNase R treatment, 2 µg of total RNA was incubated with 5 U/µL RNase R (RNR07250, Lucigen, USA) at 37 °C for 30 min before reverse transcription. For actinomycin D treatment, HCC cells were cultured in six-well plates and treated with 5 µg/mL actinomycin D (D23070, Sigma-Aldrich) when they reached approximately 60% confluence. The cells were treated for the indicated time intervals. The expression levels of circPIAS1 and linear PIAS1 mRNA were analyzed using qPCR.

### Western blotting

The cells were lysed with NETN buffer (20 mM Tris-HCl at pH 8.0, 100 mM NaCl, 1 mM EDTA, and 0.5% Nonidet P-40 (56,741, Sigma-Aldrich)) supplemented with protease and phosphatase inhibitors (Thermo Fisher Scientific). The lysate protein concentration was measured using the BCA protein assay kit (Pierce). After equalization, 10 µg of each protein sample was processed via SDS-PAGE, transferred to PVDF membranes, and blocked using 5% non-fat milk (232,100, BD Biosciences) diluted in 1× Tris-buffered saline supplemented with 0.5% Tween-20 (TBST). These membranes were incubated overnight at 4 °C with primary antibodies, followed by their HRP-labeled secondary counterparts (W4011 for rabbit and W4021 for mouse originated primary antibodies, Promega). The immunoreactive bands were visualized by the enhanced chemiluminescence (ECL). The primary antibodies were diluted with Antibody Dilution Buffer (P0023A, Beyotime) and the secondary antibodies were diluted in 1× TBST. GAPDH was used as the control. Band quantification was done via ImageJ.

### CCK-8 and colony formation assays

Cell viability was evaluated using a CCK-8 kit (CK04, Dojindo). The cells were seeded in 5 replicates in a 96-well plate at a density of 1,000 cells and cultured with 100 µL DMEM containing 10% FBS per well. At the indicated time point, 10 µL of the CCK-8 solution was added to each well, and the cells were incubated for another 2 h at 37 °C. The OD value at 450 nm was then measured using a microplate reader (ELx800, BioTek). For colony formation, HCC cells (1,000/well) were placed in six-well plates. After 14 days, cells were fixed with 4% paraformaldehyde, tinted with 0.1% crystal violet (C6158, Sigma), and left to dry. Colonies, identified as groups of 50 or more cells, were counted under a light microscope. The procedure was conducted three times.

### Wound healing assay

HCC cells were plated to full confluency on a 96-well dish, subjected to scratching, and then cultured under serum-free conditions for an additional 48 h. Images were captured at 0 and 48 h to document changes in wound width.

### Transwell assay

HCC cells (50,000 cells/well) were seeded into the upper chamber of 24-well Transwell plates with 8 μm-pore size (3374, Corning, USA) without Matrigel coating, while the lower chamber was filled with DMEM containing 10% FBS as a chemoattractant. After incubating for 24 h, non-migrating cells on the upper side of the chamber were removed by scrubbing, and migrating cells on the lower side were fixed with 4% paraformaldehyde and stained with crystal violet. The number of migrating cells was assessed in six random microscope fields.

### Measurement of Fe^2+^, lipid reactive oxygen species (ROS), and GSH levels

To evaluate lipid ROS and Fe^2+^ levels in HCC cells, C11-BODIPY581/591 (10 µM, GC40165, GLPBIO) or FerroOrange (5 µM, F374, DOJINDO) was introduced into the cell culture medium supernatant and incubated for 30 min in the dark, followed by PBS washing. The cells were then visualized using a fluorescence microscope (DMi8, Leica). GSH levels were determined using a GSH/GSSG Assay Kit (S0053, DOJINDO) following the manufacturer’s instructions.

### RNA fluorescence in situ hybridization (FISH)

The FISH assay was conducted using the Fluorescent In Situ Hybridization Kit (H0101, GenePharma, China) according to the manufacturer’s instructions. A total of 30,000 cells were seeded into confocal dishes, fixed, and then exposed to Cy3-labelled miR-455-3p and FAM-labelled circPIAS1 probes (Servicebio, China). Cell nuclei were counterstained with DAPI. Fluorescence was excited and imaged using a confocal laser scanning microscope (DMi8, Leica).

### RNA immunoprecipitation (RIP) assay

The RIP assay was conducted using the Magna RIP RNA-Binding Protein Immunoprecipitation Kit (17–700, Merck) according to the manufacturer’s instructions. In brief, the protein A/G magnetic beads were first incubated with specific antibodies and anti-IgG controls. Subsequently, cells were lysed using the kit’s lysis buffer and incubated overnight with protein A/G magnetic beads coated with antibodies. After washing, the lysates were digested with protease and RNase inhibitors for purification. The isolated RNA underwent qPCR evaluation.

### RNA pulldown assay

The RNA pull-down procedure used the RNA pull-down kit (20,164, Thermo Scientific) according to the manufacturer’s instructions. Biotinylated probes for circPIAS1 and control sequences were custom-designed and obtained from Sangon Biotech (China). Approximately 1 × 10^7^ cells were lysed in lysis buffer after washing with ice-cold PBS, followed by incubation at room temperature with 3 µg of biotinylated probes for 2 h. Subsequently, the biotin-coupled RNA complex was pulled down by incubating the cell lysates with streptavidin magnetic beads for an additional 4 h. The beads were then washed five times with lysis buffer, and bound miRNAs in the pull-down materials were extracted using Trizol reagent. A qPCR assay was conducted to analyze the bound miRNAs. The sequences of the biotin-labeled circPIAS1 and control probes are as follows: circPIAS1 probe: UCUCGAAAGCGCUGACUGUUGUCUGAUGCCAUCAAUAAUAAGGUGUUCAUAUGGAGCCUUCUU; control probe: UUGUACUACACAAAAGUACUG.

### Separation of nuclear and cytoplasm fractions

RNA extraction from the nuclear and cytoplasmic fractions was carried out using the PARIS Kit (AM1921, Life Technologies) according to the manufacturer’s instructions. Following extraction, qPCR was employed to determine the relative RNA levels from each fraction. Nuclear control transcripts U3 and U6 were utilized, with GAPDH mRNA acting as the cytoplasmic marker.

### Chromatin immunoprecipitation (ChIP) assay

ChIP assays were performed using the EZ-Magna ChIP A/G Kit (17-10086, Millipore) according to the manufacturer’s instructions. Briefly, HCC cells were cross-linked with 1% formaldehyde (F8775, Sigma-Aldrich) at room temperature for 10 min and then quenched with glycine. After washing, cells were lysed in the lysis buffer at 4 °C for 30 min and sonicated (Sonifier 450D, Branson) (50% amplitude, 10 s pulse, 30 s rest on ice, 4 cycles) to generate DNA fragments (200-1,000 bp in length). A total of 10 µg of protein-DNA complexes were immunoprecipitated with the indicated antibodies or isotype-matched IgG. The immunoprecipitated DNA was then purified and utilized for qPCR analysis. The ChIP-qPCR primer sequences are shown in Supplementary Table 1.

### In vivo animal study

All mice (Charles River Laboratories, China) were handled in compliance with the Guide for the Care and Use of Laboratory Animals and approved by the Institutional Animal Care and Use Committee of the Third Affiliated Hospital of Sun Yat-sen University. They were housed in standard, specific pathogen-free conditions with ad libitum access to rodent laboratory chow and tap water, maintaining a temperature of 24 ± 1 °C, a humidity of 50 ± 10%, and a 12:12 h light/dark cycle. Food and water were freely available throughout the study.

For in vivo tumor growth monitoring, BALB/c-nude mice aged 3–4 weeks were subcutaneously injected with 1 × 10^6^ HCC cells. Tumors were measured every two days, and on the final day, they were excised, weighed, and their volumes calculated using the formula: tumor volume = π/6 × large diameter × smaller diameter^2^.

To evaluate in vivo metastasis, BALB/c-nude mice aged 5–6 weeks were intravenously injected with 100 µL of HCC cell suspension (5 × 10^6^/mL) through the tail vein. After 8 weeks, mice from each group were euthanized, and their lung tissues were collected for the evaluation of metastatic foci using standard histopathological methods.

To assess the treatment effect of ZZW-115, 5 × 10^5^ HCC cells were subcutaneously injected into 4- to 6-week-old BALB/c-nude mice. When the tumor volumes averaged approximately 200 mm^3^, mice were randomly assigned to specified groups. Treatments were administered every five days per week, including: 0.5% DMSO in PBS (carrier), lenvatinib 30 mg/kg (oral), ZZW-115 5 mg/kg (i.p. injection), or a combination of lenvatinib 30 mg/kg and ZZW-115 5 mg/kg. Mouse weight and tumor volume were measured every five days. After 25 days, mice were euthanized, and subcutaneous tumors were excised for routine hematoxylin and eosin (H&E) staining and subsequent IHC analysis, following the manufacturer’s instructions.

### Statistical analysis

Each experiment was performed thrice for technical consistency. Error bars, unless specified otherwise, denote standard deviation (SD). Data were analyzed using GraphPad Prism 6.0, with pairwise group comparisons conducted using a two-tailed, unpaired Student’s t-test, and multiple comparisons analyzed using one-way or two-way ANOVA. Statistical significance was represented as *p < 0.05, **p < 0.01, and ***p < 0.001, with ns indicating no significance.

## Results

### Circular RNA circPIAS1 is upregulated in HCC tissues and cell lines

**Fig. 1:**
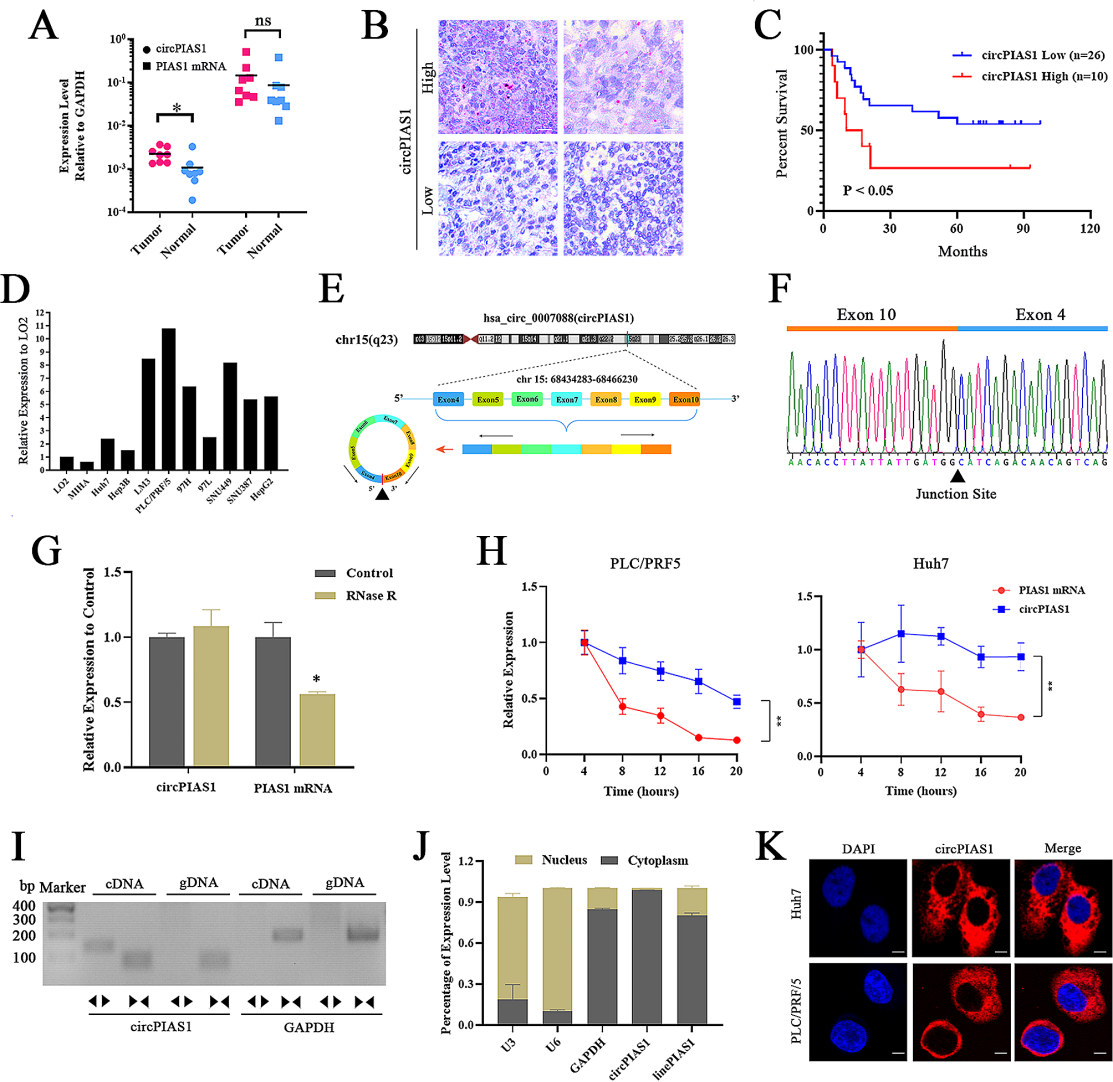
Expression and characterization of circPIAS1 in HCC. (**A**) Higher circPIAS1 expression in HCC tissues compared to normal tissues. (**B**) RNAScope assay images showing high and low circPIAS1 expression in HCC tissues. Scale bar = 200 µm. (**C**) Association of circPIAS1 expression with overall survival in HCC patients based on Kaplan-Meier analysis. (**D**) Elevated circPIAS1 expression in HCC cell lines compared to normal hepatic cells. (**E**) CircPIAS1 is derived from back-splicing of 4–10 exons of PIAS1 pre-mRNA. A black triangle indicates the“head-to-tail” splice junction site. (**F**) Confirmation of circPIAS1 back-splicing junction sequence by Sanger sequencing. (**G**) qPCR analysis of circPIAS1 and linear PIAS1 mRNA expressions with or without RNase R treatment for 30 min. (**H**) qPCR analysis of circPIAS1 and PIAS1 mRNA expressions in Huh7 and PLC/PRF/5 cells treated with actinomycin D (2 µg/mL) at the indicated time points. (**I**) RT-PCR analysis of circPIAS1 and linear PIAS1 mRNA using divergent and convergent primers in Huh7 cells. GAPDH acted as a control. (**J**) Subcellular localization of circPIAS1 in Huh7 cells assessed by qPCR analysis. (K) FISH assay demonstrating predominantly cytoplasmic localization of circPIAS1. Scale bar = 50 μm

Previous sequencing data have indicated aberrant expression of hsa_circ_0007088 (derived from the PIAS1 gene, circPIAS1) in various cancers [20–22], but its regulatory role in HCC remains unclear. To investigate the association between circPIAS1 expression and pathological features in HCC, we examined its levels in HCC tissues and cell lines. Our results revealed upregulation of circPIAS1 in fresh HCC tissues compared to adjacent non-cancerous tissues (Fig. 1A). Additionally, patients with higher circPIAS1 expression exhibited significantly lower survival rates (Fig. 1B-C). Table 1 shows a close association between circPIAS1 and TNM stage, but not with other parameters such as age, sex, tumor grade, or family history. Furthermore, circPIAS1 expression was consistently elevated in HCC cell lines compared to immortalized hepatic cells **(**Fig. 1D). CircPIAS1, located on chromosome 15 (chr15: 68,434,283–68,466,230), is derived from back-splicing of exons 4–10 of the host gene PIAS1, with a mature sequence length of 700 bp (Fig. 1E). Sanger sequencing confirmed the back-splicing site of circPIAS1 (Fig. 1F). Stability analysis using RNase R or actinomycin D treatment revealed that the circular form (circPIAS1) was more resistant to RNase R compared to the linear form (PIAS1 mRNA), which significantly degraded (Fig. 1G). Additionally, the half-life of circPIAS1 was longer than that of linear PIAS1 mRNA after treatment with actinomycin D (Fig. 1H), indicating greater stability of circPIAS1 than PIAS1 mRNA. Moreover, to exclude genomic rearrangement of the host gene, convergent primers for PIAS1 mRNA and divergent primers for circPIAS1 were designed. CircPIAS1 was only amplified by divergent primers in cDNA but not in genomic DNA, confirming the presence of circularized PIAS1 exons and excluding trans-splicing products (Fig. 1I). Nucleocytoplasmic fractionation and FISH assays showed that circPIAS1 predominantly existed in the cytoplasm of HCC cells (Fig. 1J-K). These data suggest that circPIAS1 expression may play a role in HCC progression and patient prognosis.

**Table 1:** Association of circPIAS1 expression and clinical information in 36 HCC patients

Clinical features	Low circPIAS1	High circPIAS1	*P* Value
Number	*n* = 26	*n* = 10	
Sex			0.096
Male	20	10	
Female	6	0	
Age (yeas)			0.293
<55	16	8	
≥ 55	10	2	
Tumor size (cm)			0.667
<5	7	2	
≥ 5	19	8	
Lymph node involvement			0.676
Yes	4	1	
No	22	9	
Tumor grade			0.293
I	2	2	
II-III	24	8	
TNM stage			0.035*
I + II	20	4	
III + IV	6	6	
HBV infection			0.321
Yes	22	7	
No	4	3	
AFP			0.137
<400	15	3	
≥ 400	11	7	

### CircPIAS1 knockdown inhibits the proliferation and migration of HCC cells in vitro and in vivo

**Fig. 2:**
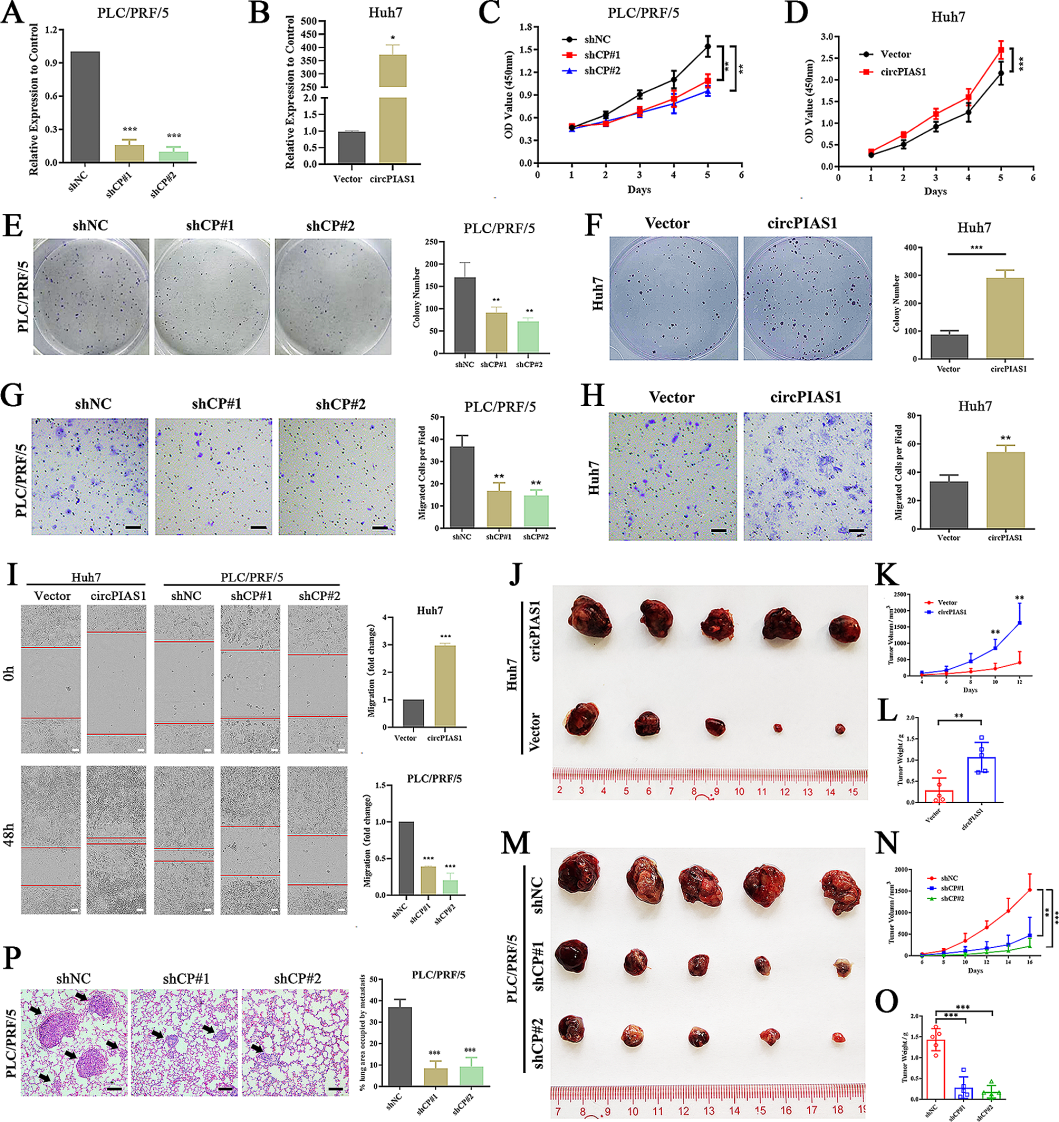
CircPIAS1 promotes HCC cell proliferation and migration in vitro and in vivo. (**A**) qPCR analysis of circPIAS1 expression in PLC/PRF/5 cells treated with circPIAS1 shRNAs. (**B**) qPCR analysis of circPIAS1 expression in Huh7 cells after circPIAS1 overexpression. (**C**-**D**) CCK-8 assays assessing cell proliferation in circPIAS1 knockdown (**C**) or overexpression (**D**) HCC cells. (**E**-**F**) Plate colony formation assays in circPIAS1 knockdown (**E**) or overexpression (**F**) HCC cells. Left: representative images; Right: quantification of colony number. (**G**-**H**) Transwell assays measuring migration in circPIAS1 knockdown (**G**) or overexpression (**H**) HCC cells. Left: representative images; Right: quantification of migratory cell number. (**I**) Wound-healing assays in circPIAS1 knockdown or overexpression HCC cells. Left: representative images; Right: quantification of scratch width distance. The red line indicates the scratch edge. (**J**) Subcutaneous tumor xenografts from control and circPIAS1 overexpression groups. (**K**) Tumor growth curves of control and circPIAS1 overexpression xenografts. (**L**) Tumor weights of control and circPIAS1 overexpression xenografts at the end point. (**M**) Subcutaneous tumor xenografts from control and sh-circPIAS1 groups. (**N**) Tumor growth curves of control and sh-circPIAS1 xenografts. (**O**) Tumor weights of control and sh-circPIAS1 xenografts at the end point. (**P**) Left: representative images of H&E staining of lung metastasis loci. Right: percentage of net lung area occupied by metastases quantified for each group. Scale bar = 200 μm. shCP, sh-circPIAS1

To explore the role of circPIAS1 in HCC development, we designed two shRNAs to silence circPIAS1 effectively in PLC/PRF/5 cells. Conversely, we used a lentivirus infection system to upregulate circPIAS1 in Huh7 cells (Fig. 2A-B). Viability and proliferation assays using CCK-8 and colony formation showed that circPIAS1 knockdown suppressed the viability and proliferation of HCC cells, whereas circPIAS1 overexpression significantly increased cell viability and proliferation (Fig. 2C-F). Migration assays including wound healing and transwell assays demonstrated that circPIAS1 depletion inhibited the migratory ability of HCC cells, and vice versa (Fig. 2G-I). To investigate the role of circPIAS1 in tumor progression in vivo, we subcutaneously implanted circPIAS1 knockdown or overexpression HCC cells into nude mice and monitored the growth of xenograft tumors. Our analysis revealed that circPIAS1 overexpression notably enhanced the growth rate and tumor weight compared to the control group (Fig. 2J-L). Conversely, reduced tumor size and weight were observed in sh-circPIAS1 mice compared to sh-NC mice at the same endpoint (Fig. 2M-O). Additionally, we used a tail vein injection model to assess the migratory ability of HCC cells in vivo. The results indicated a significant reduction in the area occupied by lung metastases after depletion of circPIAS1 expression compared to the control group (Fig. 2P). These data suggest that circPIAS1 contributes to the aggressive phenotypes of HCC cells both in vitro and in vivo.

### CircPIAS1 inhibits ferroptosis in HCC cells

**Fig. 3:**
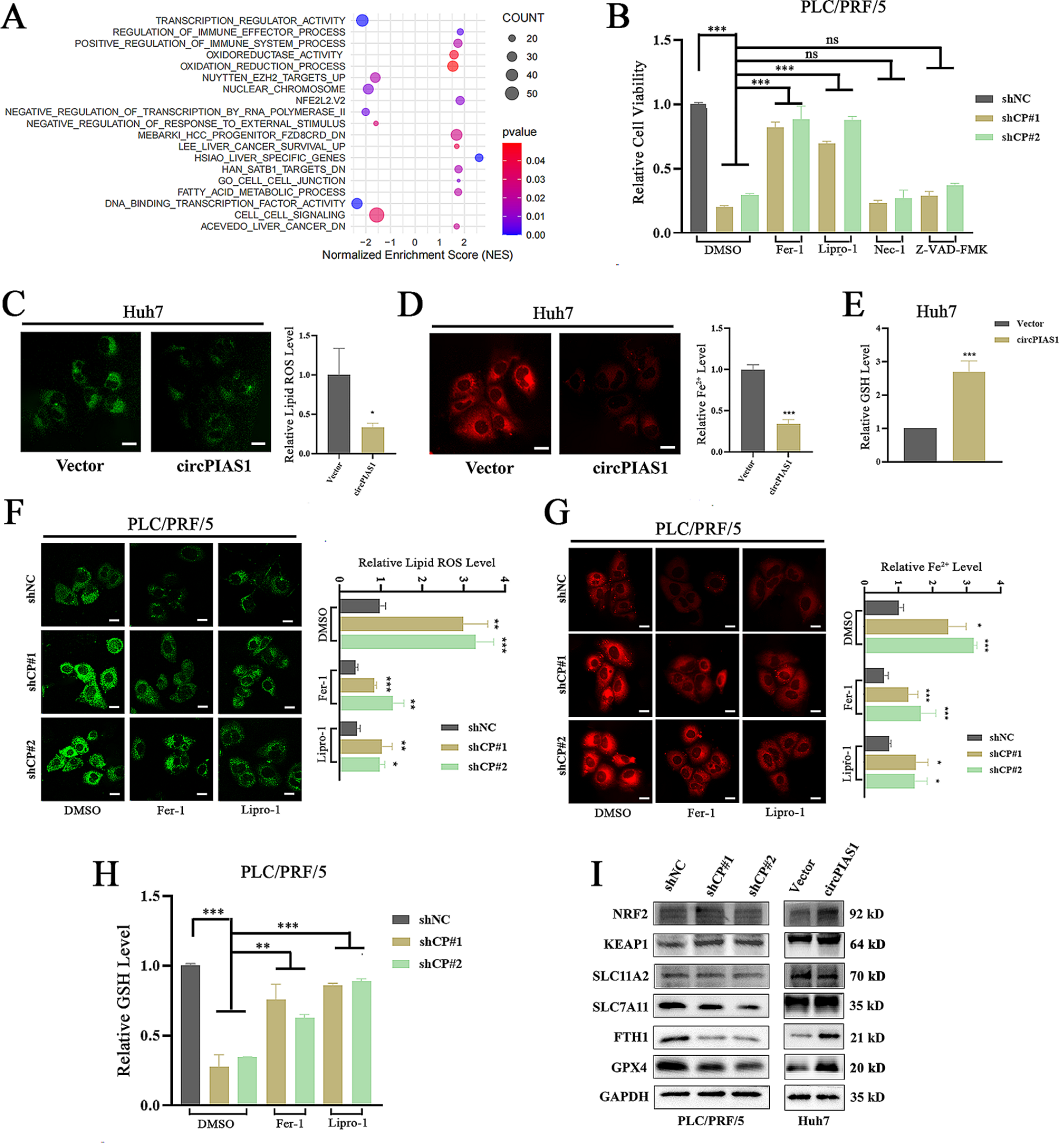
CircPIAS1 inhibits ferroptosis in HCC cells. (**A**) GSEA result of differentially expressed gene pathways affected by circPIAS1 depletion in HCC cells. (**B**) Viability analysis of sh-circPIAS1 HCC cells treated with Z-VAD-FMK, Nec-1, Lipro-1, or Fer-1 for 24 h. (**C**) Measurement of lipid ROS levels using C11-BODIPY581/591 probe in circPIAS1 overexpression cells. Left: representative images; Right: quantification of lipid ROS level. (**D**) Detection of intracellular Fe^2+^ levels with FerroOrange probe in circPIAS1 overexpression cells. Left: representative images; Right: quantification of Fe^2+^ level. (**E**) Measurement of GSH levels using a GSH and GSSG Assay Kit in circPIAS1 overexpression cells. (**F**) Assessment of lipid ROS levels using C11-BODIPY581/591 probe in sh-circPIAS1 cells with or without Fer-1 or Lipro-1 treatment. Left: representative images; Right: quantification of lipid ROS level. (**G**) Detection of intracellular Fe^2+^ levels with FerroOrange probe in sh-circPIAS1 cells with or without Fer-1 or Lipro-1 treatment. Left: representative images; Right: quantification of Fe^2+^ level. (**H**) Measurement of GSH levels using a GSH and GSSG Assay Kit in sh-circPIAS1 cells with or without Fer-1 or Lipro-1 treatment. (**I**) Western blotting analysis of NUPR1, FTH1, KEAP1, SLC11A2, GPX4, SLC7A11, and NRF2 protein levels in circPIAS1 knockdown or overexpression HCC cells. Scale bar = 20 μm. shCP, sh-circPIAS1

To elucidate the mechanism underlying circPIAS1-mediated tumor promotion, we conducted RNA sequencing in circPIAS1-depleted HCC cells. GSEA revealed that the differentially expressed genes were predominantly enriched in pathways related to oxidation-reduction processes, oxidoreductase activity, fatty acid metabolic processes, and the NFE2L2.V2 pathway, indicating that circPIAS1 depletion is involved in signaling pathways related to oxidative metabolism-associated cell death (Fig. 3A). To determine the type of cell death induced by circPIAS1 knockdown, sh-circPIAS1 HCC cells were treated with inhibitors of apoptosis (Z-VAD-FMK), necroptosis (necrostatin-1, Nec-1), and ferroptosis (ferrostatin-1, Fer-1, and liproxstatin-1, Lipro-1). Our results showed that the growth inhibition caused by circPIAS1 depletion was significantly reversed by Fer-1 and Lipro-1, indicating that circPIAS1 depletion induces ferroptosis in HCC cells (Fig. 3B). We further investigated the effects of circPIAS1 expression on ferroptosis. Overexpression of circPIAS1 reduced intracellular Fe^2+^ and lipid ROS levels, while increasing GSH content (Fig. 3C-E). Conversely, in sh-circPIAS1 HCC cells, intracellular Fe^2+^ and lipid ROS levels increased, and GSH levels decreased, which could be reversed by Fer-1 or Lipro-1 treatment (Fig. 3F-H). Additionally, we examined the levels of ferroptosis-related proteins in circPIAS1-overexpressing or knockdown HCC cells. The results demonstrated that the protein levels of SLC7A11, FTH1, and GPX4 decreased after circPIAS1 depletion and increased significantly following circPIAS1 overexpression (Fig. 3I). These results suggest that circPIAS1 expression can suppress ferroptosis activity in HCC cells.

### NUPR1 is a downstream factor mediating circPIAS1–regulated ferroptosis in HCC cells

**Fig. 4:**
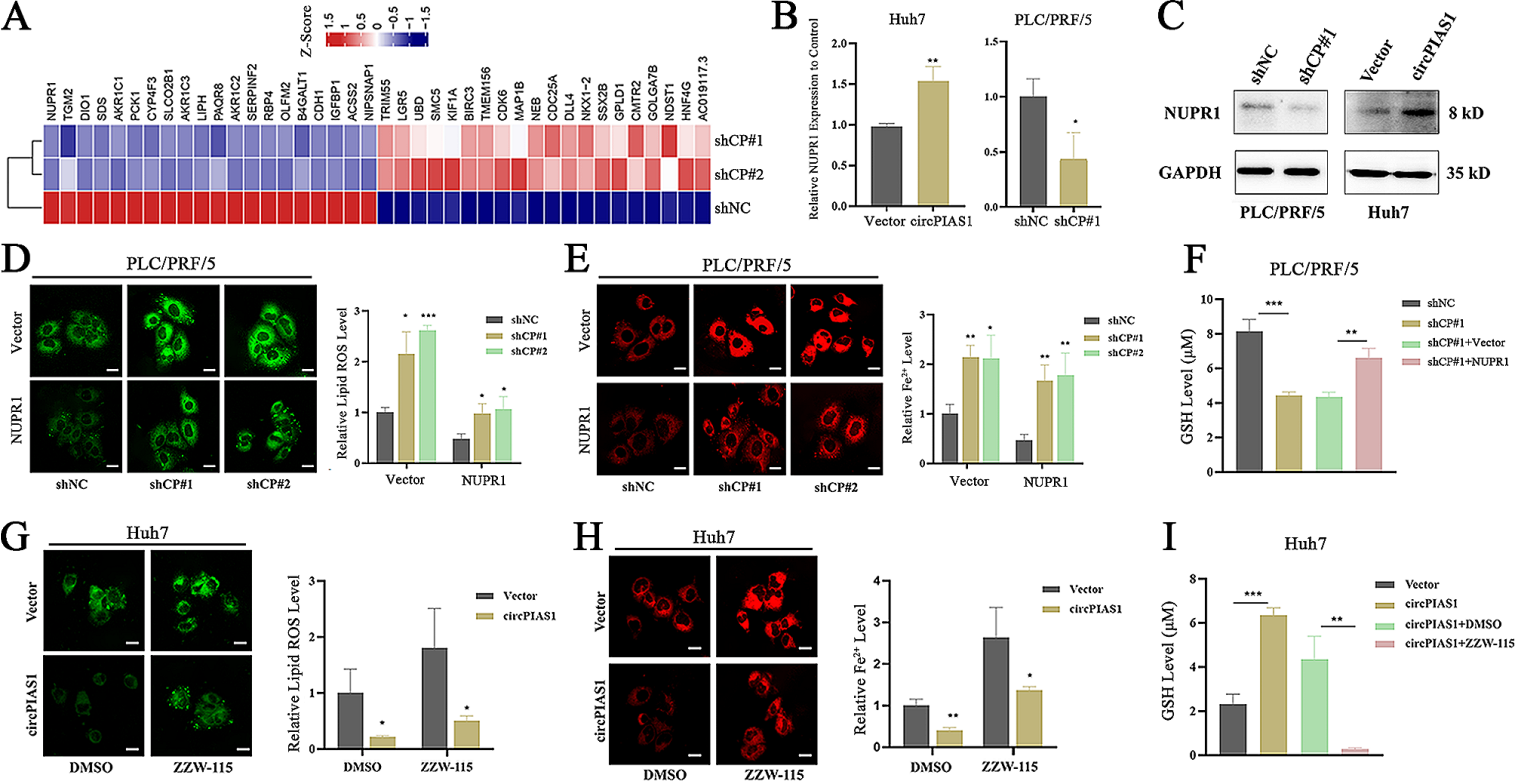
CircPIAS1 regulates the sensitivity of HCC cells to ferroptosis through NUPR1 (**A**) Heatmap showing the top 20 differentially expressed genes in sh-circPIAS1 PLC/PRF/5 cells. Genes with fold change > 2 and *p* < 0.05 were considered significantly different. (**B**-**C**) Expression of NUPR mRNA and protein in circPIAS1 overexpression or knockdown HCC cells confirmed by qPCR analysis (**B**) or western blotting analysis (**C**). (**D**) Lipid ROS levels in sh-circPIAS1 cells with or without NUPR1 overexpression. Left: representative images; Right: quantification of lipid ROS level. (**E**) Intracellular Fe^2+^ levels in sh-circPIAS1 cells with or without NUPR1 overexpression. Left: representative images; Right: quantification of Fe^2+^ level. (**F**) GSH levels in sh-circPIAS1 cells with or without NUPR1 overexpression. (**G**) Lipid ROS levels in circPIAS1 overexpression cells with or without ZZW-115 treatment. Left: representative images; Right: quantification of lipid ROS level. (**H**) Intracellular Fe^2+^ levels in circPIAS1 overexpression cells with or without ZZW-115 treatment. Left: representative images; Right: quantification of Fe^2+^ level. (**I**) GSH levels in circPIAS1 overexpression cells with or without ZZW-115 treatment. Scale bar = 20 μm. shCP, sh-circPIAS1

Based on the fold-change and p-value filtering of the differential genes from the aforementioned RNA sequencing data, NUPR1 emerged as the most downregulated gene following circPIAS1 depletion (Fig. 4A). NUPR1 is known as a key regulator of ferroptosis [17, 19]. Therefore, we aimed to investigate whether circPIAS1-mediated regulation of ferroptosis in HCC cells relies on NUPR1 expression. qPCR and western blotting analyses revealed that circPIAS1 silencing decreased NUPR1 protein and mRNA levels, whereas circPIAS1 overexpression increased NUPR1 levels in PLC/PRF/5 and Huh7 cells, respectively (Fig. 4B-C). The increase in intracellular Fe^2+^ and lipid ROS levels, or the decrease in GSH levels induced by circPIAS1 depletion, could be reversed by forced expression of NUPR1 in HCC cells (Fig. 4D-F). Conversely, circPIAS1-overexpressing HCC cells treated with the NUPR1 inhibitor, ZZW-115, exhibited a ferroptotic phenotype compared to the control (Fig. 4G-I). Furthermore, cell proliferation and migration abilities were rescued by NUPR1 overexpression in sh-circPIAS1 HCC cells (Supplementary Fig. 1A-C), while administration of ZZW-115 partially mitigated the aggressive phenotypes induced by circPIAS1 overexpression, as revealed by functional assays (Supplementary Fig. 1D-F). These results suggest that circPIAS1 regulates ferroptosis in HCC cells through modulation of NUPR1 expression.

### CircPIAS1 regulates NUPR1 expression by sponging mir-455-3p in HCC cells

**Fig. 5:**
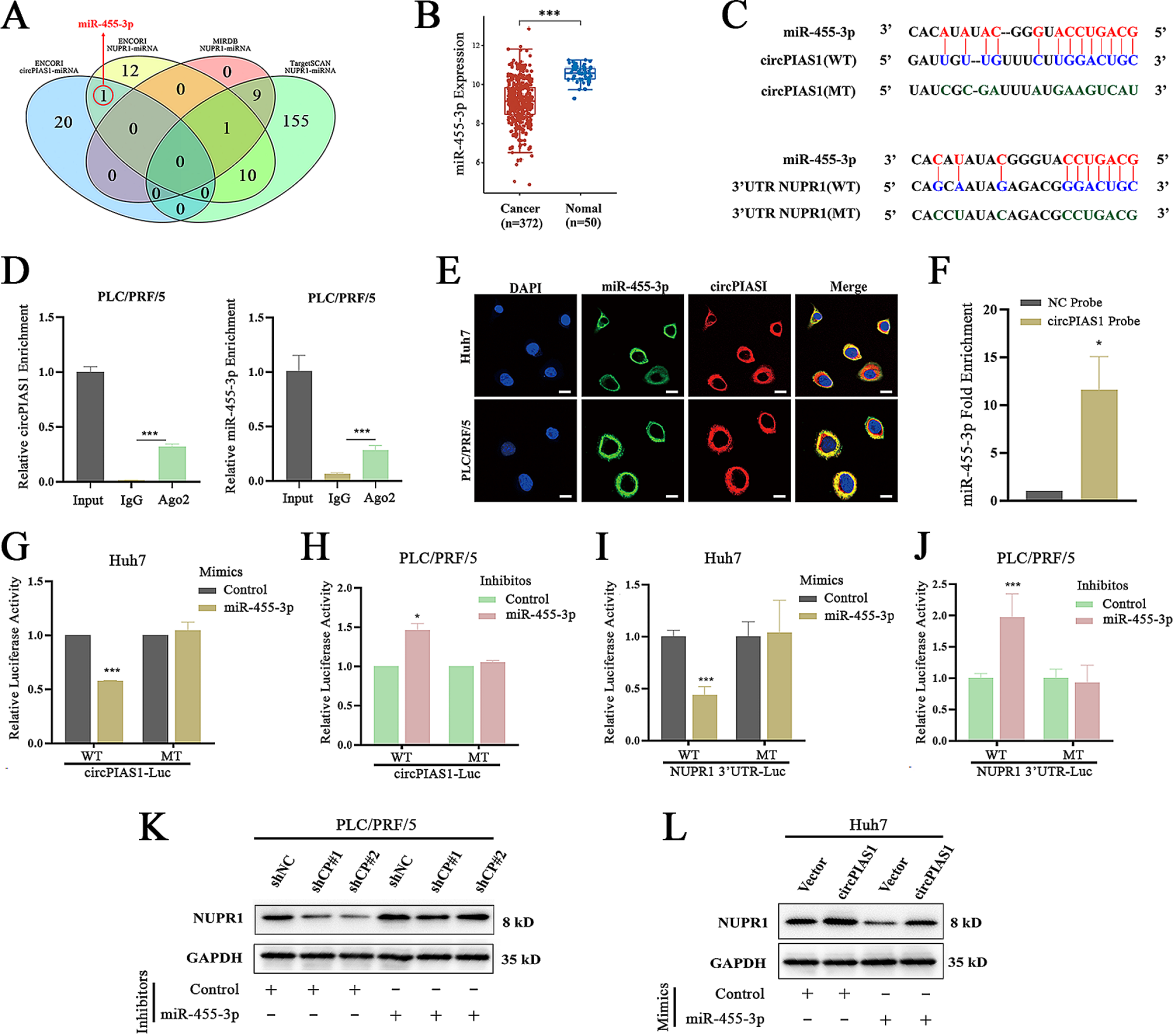
CircPIAS1 regulates NUPR1 expression by sponging miR-455-3p in HCC cells. (**A**) Venn diagram depicting potential interacting miRNAs of circPIAS1 and NUPR1 3’ UTR. (**B**) Expression of miR-455-3p in HCC tumor and normal tissues from the GEPIA database. (**C**) Upper: Predicted binding sites between miR-455-3p and circPIAS1; Lower: Putative binding sequences of miR-455-3p on NUPR1 3’ UTR. (**D**) RIP assays showing the binding of circPIAS1 (left) and miR-455-3p (right) to the Ago2 complex in PLC/PRF/5 cells. (**E**) FISH assays revealing the co-localization of miR-455-3p and circPIAS1 in Huh7 and PLC/PRF/5 cells. Scale bar = 20 μm. (**F**) RNA pull-down assay demonstrating the enrichment of miR-455-3p in Huh7 cell lysates using a specific biotin-labeled circPIAS1 probe. (**G**-**H**) Dual luciferase reporter assays in HCC cells co-transfected with wild-type or mutant circPIAS1 reporter and miR-455-3p mimics (**G**) or inhibitor (**H**). (**I**-**J**) Dual luciferase reporter assays in HCC cells co-transfected with wild-type or mutant NUPR1 3’ UTR reporter and miR-455-3p mimics (**I**) or inhibitor (**J**). (**K**-**L**) NUPR1 protein expression in sh-circPIAS1 cells with or without transfection of miR-455-3p inhibitor (**K**), or in circPIAS1 overexpression cells with or without transfection of miR-455-3p mimics (**L**). WT, wild-type; MT, mutant; shCP, sh-circPIAS1

Given the cytoplasmic localization of circPIAS1 in HCC cells, we investigated whether circPIAS1 regulates NUPR1 expression through a ceRNA mechanism. To do so, we utilized three databases (ENCORI, TargetScan, and miRDB) to predict potential miRNAs binding to both circPIAS1 and NUPR1 mRNA. We identified miR-455-3p as a candidate because it was the only miRNA shown to interact with both circPIAS1 and NUPR1 (Fig. 5A). Furthermore, reduced expression of miR-455-3p was observed in liver tumor samples from the TCGA cohort, exhibiting an expression pattern opposite to that of circPIAS1 (Fig. 5B and Supplementary Fig. 2A-B). The binding sites of miR-455-3p with circPIAS1 and NUPR1 3’ untranslated region (3’ UTR) are shown in Fig. 5C, and the corresponding wild-type and mutant luciferase reporter plasmids of circPIAS1 and NUPR1 were constructed based on these sequences. Ago2-mediated RIP assays revealed specific pull-down of both endogenous circPIAS1 and miR-455-3p by anti-Ago2 antibody in HCC cells (Fig. 5D and Supplementary Fig. 2C-D). FISH assays also demonstrated co-localization of circPIAS1 and miR-455-3p in the cytoplasm of HCC cells (Fig. 5E). Additionally, endogenous miR-455-3p was significantly enriched by the biotinylated circPIAS1 probe compared to the negative control probe in HCC cells (Fig. 5F). Luciferase reporter assays showed that miR-455-3p mimic suppressed the activity of wild-type circPIAS1 and NUPR1 3’ UTR reporters, while the miR-455-3p inhibitor induced their activities, with no effects on the mutant counterparts (Fig. 5G-J). MiR-455-3p mimics effectively reduced the increased NUPR1 levels after circPIAS1 overexpression, whereas the miR-455-3p inhibitor significantly offset the decreased NUPR1 expression in sh-circPIAS1 HCC cells (Fig. 5K-L). These data suggest that circPIAS1 regulates NUPR1 expression by acting as a ceRNA for miR-455-3p in HCC cells.

### CircPIAS1 promotes the aggressive phenotypes of HCC cells by sponging miR-455-3p

**Fig. 6:**
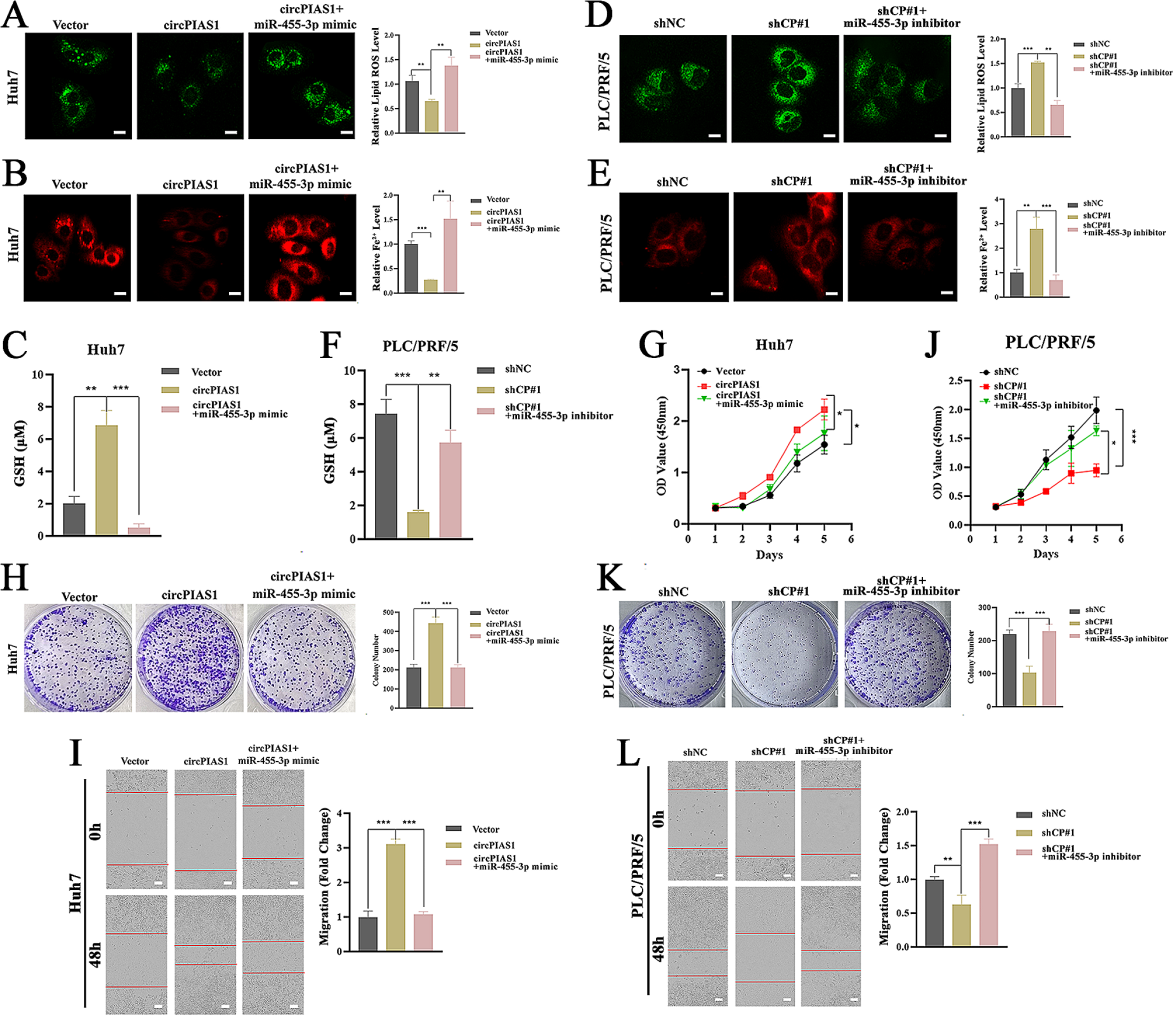
MiR-455-3p reverses circPIAS1-induced ferroptosis inhibition and aggressive phenotypes in HCC cells. (**A**) Lipid ROS levels in circPIAS1 overexpression cells with or without miR-455-3p mimic transfection. Left: representative images; Right: quantification of lipid ROS level. (**B**) Intracellular Fe^2+^ levels in circPIAS1 overexpression cells with or without miR-455-3p mimic transfection. Left: representative images; Right: quantification of Fe^2+^ level. (**C**) GSH levels in circPIAS1 overexpression cells with or without miR-455-3p mimic transfection. (**D**) Lipid ROS levels in sh-circPIAS1 cells with or without miR-455-3p inhibitor transfection. Left: representative images; Right: quantification of lipid ROS level. (**E**) Intracellular Fe^2+^ levels in sh-circPIAS1 cells with or without miR-455-3p inhibitor transfection. Left: representative images; Right: quantification of Fe^2+^ level. (**F**) GSH levels in sh-circPIAS1 cells with or without miR-455-3p inhibitor transfection. (**G**-**I**) Cell proliferation (**G**), colony formation (**H**), and wound-healing (**I**) abilities assessed in circPIAS1 overexpression cells with or without miR-455-3p mimic transfection. (**J**-**L**) Cell proliferation (**J**), colony formation (**K**), and wound-healing (**L**) abilities assessed in sh-circPIAS1 cells with or without miR-455-3p inhibitor transfection. The red line indicates the scratch edge. Scale bar = 20 μm. shCP, sh-circPIAS1

To ascertain whether circPIAS1’s tumor-promoting effects are mediated through its sponge effect on miR-455-3p, we conducted rescue experiments in HCC cells. We found that the circPIAS1 overexpression-induced decrease in intracellular Fe^2+^ and lipid ROS, as well as the increase in GSH levels, were rescued by introducing a miR-455-3p mimic (Fig. 6A-C). Conversely, the circPIAS1 silence-induced accumulation of intracellular Fe^2+^ and lipid ROS, and reduction in GSH levels, were counteracted by transfection with a miR-455-3p inhibitor (Fig. 6D-F). Furthermore, we demonstrated that the enhanced proliferation and migration capacities of circPIAS1-overexpressing Huh7 cells were significantly abrogated by the introduction of a miR-455-3p mimic (Fig. 6G-I), while the suppressive effects on proliferation and migration capacities of sh-circPIAS1 PLC/PRF/5 cells were reversed after transfection with a miR-455-3p inhibitor (Fig. 6J-L). These data suggest that circPIAS1 promotes the aggressiveness of HCC cells by acting as a molecular sponge for miR-455-3p.

Furthermore, to elucidate the direct regulation of ferroptosis by miR-455-3p in HCC cells, we established HCC cell lines with stable overexpression or depletion of miR-455-3p. The expression level of miR-455-3p was verified by qPCR assays (Supplementary Fig. 3A). MiR-455-3p overexpression enhanced the ferroptosis phenotype of HCC cells, as evidenced by increased intracellular Fe^2+^ and lipid ROS levels, and decreased GSH levels, which could be reversed by forced expression of NUPR1 (Supplementary Fig. 3B-D). Conversely, the opposite effects were observed in miR-455-3p-depleted HCC cells when treated with ZZW-115 (Supplementary Fig. 3E-G). Functional assays demonstrated that NUPR1 could also rescue the inhibitory effect on proliferation and migration caused by miR-455-3p overexpression in HCC cells (Supplementary Fig. 4A-C), and the promoting effect on cell proliferation and migration by depletion of miR-455-3p was reversed by ZZW-115 administration (Supplementary Fig. 4D-F). Moreover, through qPCR and western blotting analyses, we showed that levels of NUPR1 protein and mRNA were decreased in miR-455-3p-overexpressing cells and increased in miR-455-3p-depleted cells (Supplementary Fig. 4G-H). These results indicate that NUPR1 acts as a direct downstream target of miR-455-3p in HCC cells.

### NUPR1 promotes the transcription of FTH1 by binding to its promoter region

**Fig. 7:**
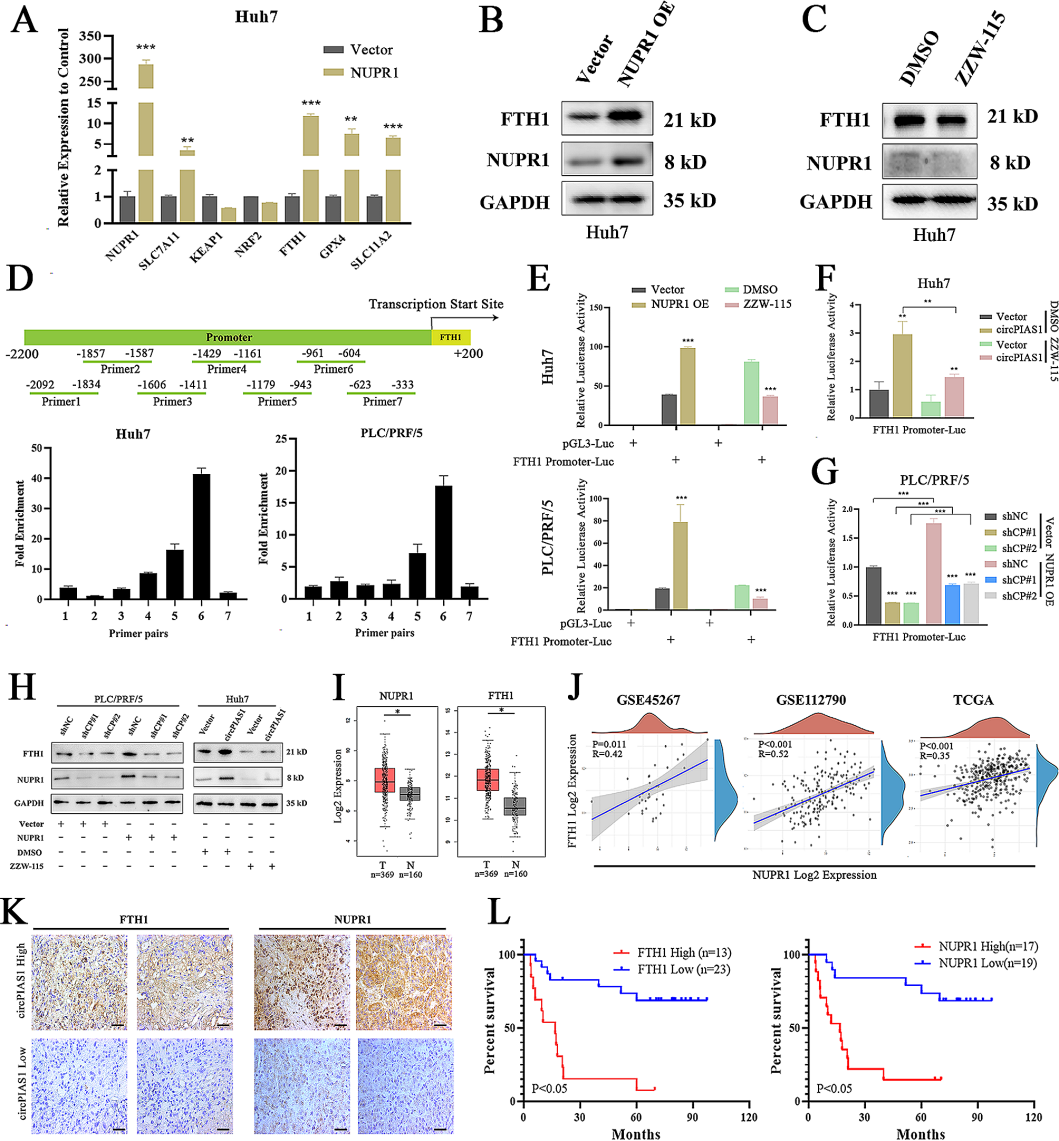
NUPR1 promotes the transcription of FTH1 by binding to its promoter region. (**A**) qRT-PCR analyses of NUPR1, FTH1, KEAP1, SLC11A2, GPX4, SLC7A11, and NRF2 expression in Huh7 cells after stable NUPR1 transfection. (**B**-**C**) Western blotting analyses of NUPR1 and FTH1 protein levels in Huh7 cells with NUPR1 overexpression (**B**) or ZZW-115 treatment (**C**). (**D**) ChIP-qPCR analysis of NUPR1 protein enrichment at the FTH1 gene promoter region. Upper panel: qPCR primer pairs covering the FTH1 gene promoter region; Lower panel: NUPR1 enrichment at the FTH1 gene promoter region. (**E**) Luciferase activity of the FTH1 promoter reporter in HCC cells with NUPR1 overexpression or ZZW-115 treatment. (**F**) Luciferase activity of the FTH1 promoter reporter in circPIAS1 overexpression cells with or without ZZW-115 treatment. (**G**) Luciferase activity of the FTH1 promoter reporter in sh-circPIAS1 cells with or without NUPR1 forced expression. (**H**) Western blotting analyses of NUPR1 and FTH1 protein levels in HCC cells under different treatments. (**I**) Upregulation of NUPR1 and FTH1 mRNA expression in tumors compared to normal controls from TCGA-HCC dataset. (**J**) Spearman’s correlations between NUPR1 and FTH1 mRNA expressions from GEPIA and GEO HCC datasets. (**K**) IHC analysis evaluating correlated FTH1 and NUPR1 protein expression with circPIAS1 in HCC tissues (*n* = 36). Scale bar = 200 μm. (**L**) Kaplan-Meier analysis of overall survival based on NUPR1 and FTH1 expression levels in HCC patients. shCP, sh-circPIAS1

Intracellular iron levels are regulated by its uptake, storage, release, and metabolism. Ferritin, comprising ferritin light chain (FTL) and ferritin heavy chain 1 (FTH1), serves as a key iron storage protein [32]. To elucidate how NUPR1 regulates the expression of ferroptosis-related proteins, HCC cells were transfected with a plasmid encoding NUPR1, resulting in a significant upregulation of FTH1 mRNA levels (Fig. 7A and Supplementary Fig. 5A). Western blotting confirmed the increase in FTH1 protein levels with NUPR1 overexpression and their decrease with ZZW-115 treatment (Fig. 7B-C and Supplementary Fig. 5B-C). Given NUPR1’s role as a transcriptional regulator, we investigated whether FTH1 is a direct target of NUPR1 in HCC cells using luciferase reporter and ChIP assays. These assays revealed significant enrichment of NUPR1 at the promoter region of the FTH1 gene locus, specifically between 500 and 1000 bp upstream of the transcription start site (Fig. 7D). The luciferase activity of the FTH1 promoter was enhanced by NUPR1 overexpression and reduced by ZZW-115 treatment in HCC cells (Fig. 7E). Furthermore, the increased luciferase activity of the FTH1 promoter in circPIAS1-overexpressing cells was counteracted by ZZW-115 treatment, while NUPR1 overexpression rescued the inhibitory effect on FTH1 promoter luciferase activity in sh-circPIAS1 cells (Fig. 7F-G). Consistent with these findings, western blotting confirmed that the decrease in FTH1 protein expression in circPIAS1 knockdown PLC/PRF/5 cells could be counteracted by NUPR1 upregulation, and the increase in FTH1 protein expression in Huh7 cells could be reversed by ZZW-115 treatment (Fig. 7H). Furthermore, bioinformatics analyses using publicly available gene expression datasets revealed upregulation of both FTH1 and NUPR1 mRNA expression in HCC tumor groups compared to normal groups, with a significant positive correlation between NUPR1 and FTH1 mRNA expressions in these HCC datasets (Fig. 7I-J). IHC analysis using HCC tissue slices showed a positive correlation between the protein expression of NUPR1 and FTH1 with circPIAS1 levels in HCC tumors (Fig. 7K; Table 2). Additionally, high expression of NUPR1 or FTH1 was associated with reduced overall survival of HCC patients (Fig. 7L). These findings indicate that NUPR1 blocks ferroptosis by inducing the expression of the iron storage protein FTH1 in HCC cells.

**Table 2:** Association of NUPR1 and FTH1 expressions with circPIAS1 level in 36 HCC specimens

Variable	*n*	circPIAS1 expression		*P*	*R*
		High	Low		
**NUPR1**					
High	17	8	9	0.025 *	0.407
Low	19	2	17		
**FTH1**					
High	13	7	6	0.018 *	0.438
Low	23	3	20		

### ZZW-115 induces ferroptosis in vivo and enhances HCC sensitivity to lenvatinib

**Fig. 8:**
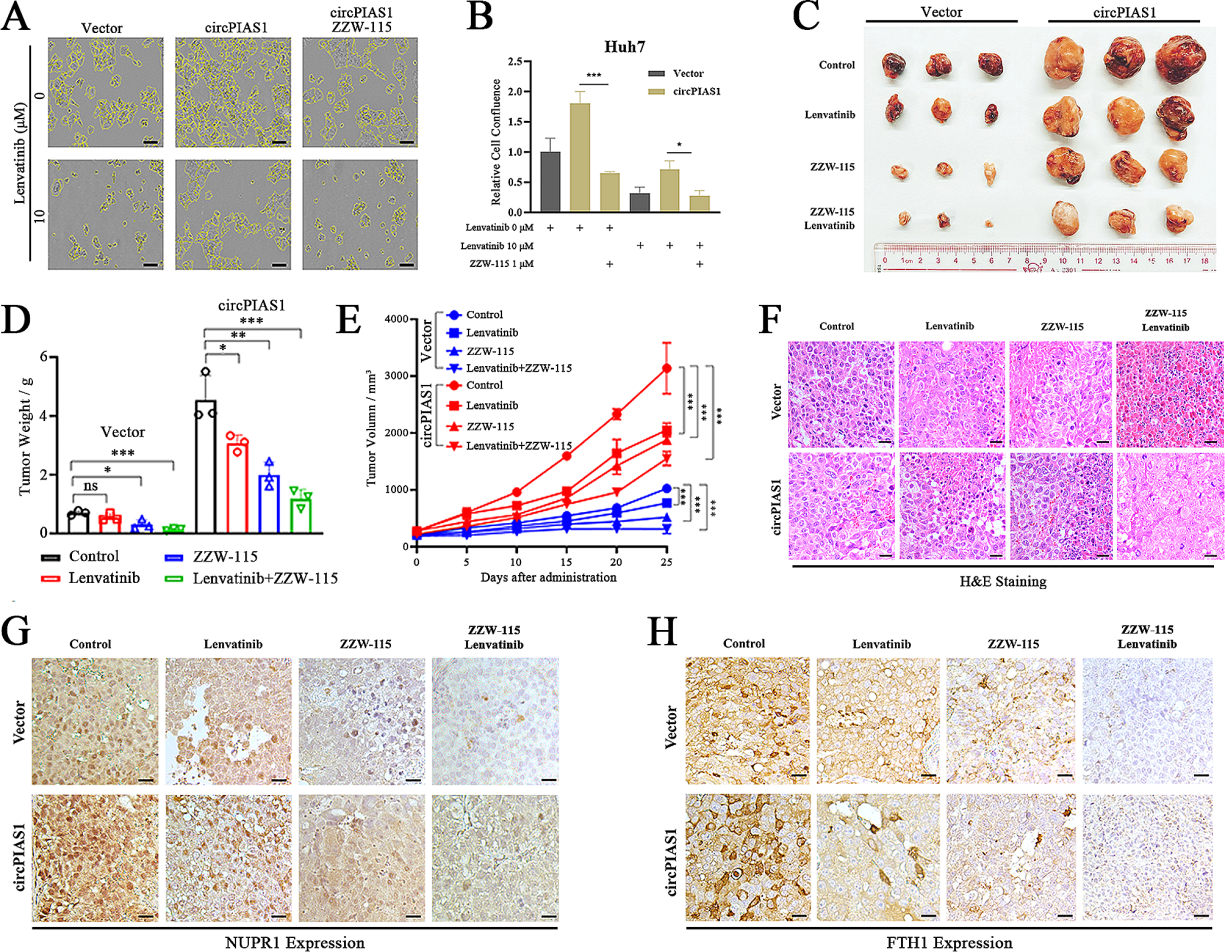
ZZW-115-induced ferroptosis enhances the sensitivity of HCC cells to lenvatinib. (**A**) CircPIAS1 overexpression HCC cells were treated with lenvatinib, ZZW-115 alone, or in combination for 36 h, and cell growth was monitored using a live-cell imaging system. Left: representative images of cells at 36 h post-treatment, with cell boundaries marked with yellow lines. (**B**) Quantification of the percentage of cell confluence in (**A**). (**C**-**E**) Subcutaneous tumor xenografts (**C**), tumor weights (**D**), and tumor growth curves (**E**) at the endpoint from control and circPIAS1 overexpression groups treated with lenvatinib, ZZW-115 alone, or in combination. (**F**) Representative images of H&E staining of the tumor xenografts. (**G**-**H**) IHC analysis of NUPR1 (**G**) and FTH1 (H) levels in the tumor xenografts. Scale bar = 200 μm. shCP, sh-circPIAS1

Chemoresistance frequently hampers the treatment of HCC patients, and recent studies have highlighted the potential of inducing ferroptosis to enhance tumor cell sensitivity to therapeutic agents. We aimed to investigate whether inhibiting NUPR1 activity could augment the sensitivity of HCC cells to lenvatinib, a first-line targeted drug for HCC. In vitro cell viability assays demonstrated that the combination of ZZW-115 and lenvatinib had a more pronounced inhibitory effect on circPIAS1-overexpressing HCC cells compared to either drug alone (Fig. 8A-B). Moreover, this synergistic effect was evident in xenograft tumors, where co-administration of ZZW-115 and lenvatinib led to the most significant suppression of tumor growth and weight in both control and circPIAS1 overexpression groups (Fig. 8C-E). Histological examination via H&E staining and IHC analysis revealed that the combined treatment induced a larger area of necrotic tissue in xenograft tumors and reduced the expression levels of NUPR1 and FTH1, respectively (Fig. 8F-H). These data suggest that ZZW-115 may enhance the efficacy of HCC treatment when used in combination with lenvatinib.

## Discussion

**Fig. 9:**
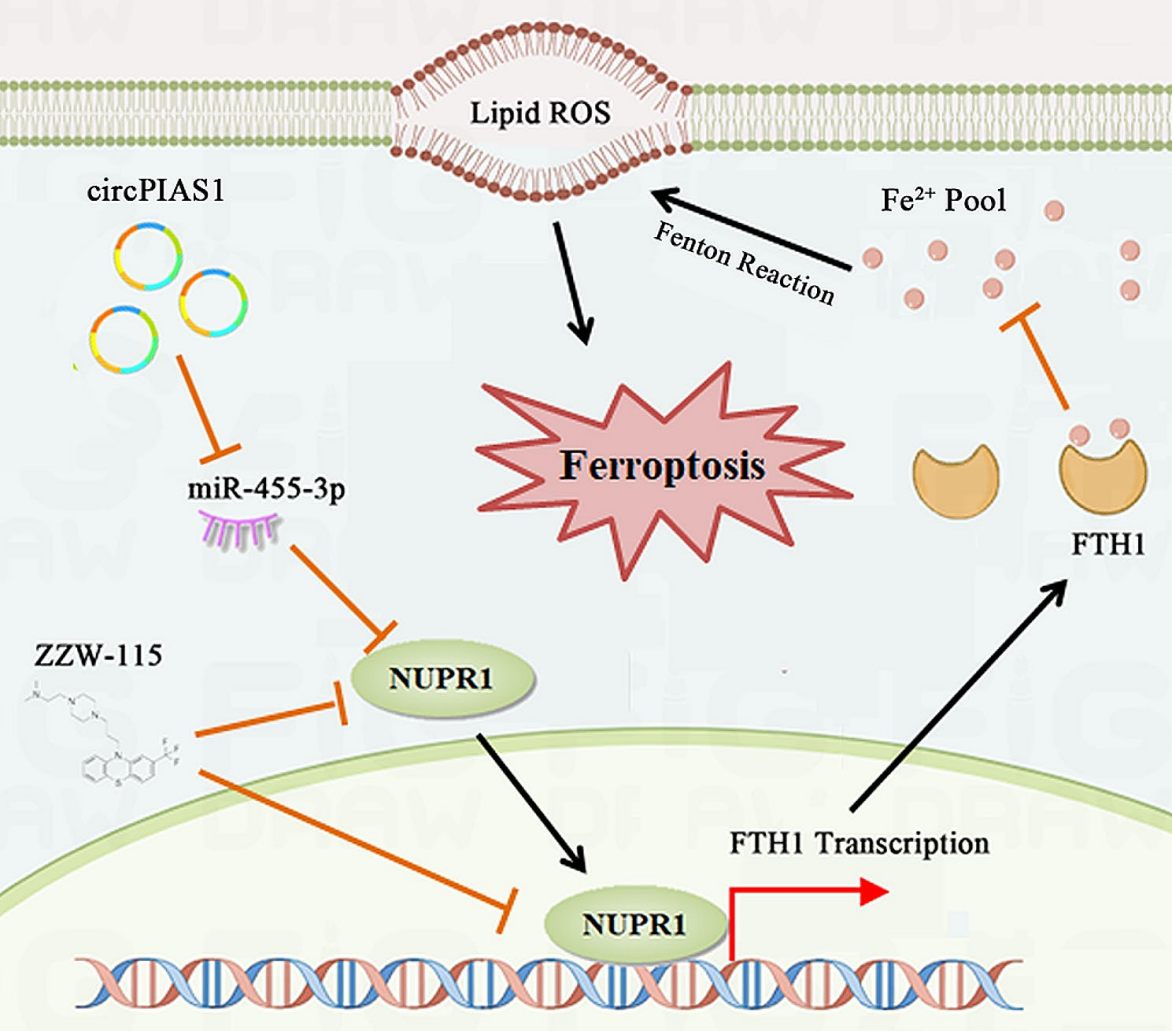
Schematic diagram depicting the tumor-promoting effects of circPIAS1 in HCC

CircRNAs, previously considered splicing “byproducts” or “junk” [23], are now known for their significant biological roles, attributed to their inherent stability from a unique structure. High-throughput RNA sequencing and circRNA-focused bioinformatics have uncovered numerous circRNAs in cells, tissues, and organisms, some implicated in tumorigenesis and cancer progression [24, 25]. Here, we identified a novel circRNA, circPIAS1, derived from exons 4 to 10 of the PIAS1 gene through back-splicing. Validation of circPIAS1’s circularization involved Sanger sequencing and treatments with actinomycin D and RNase R to confirm its head-to-tail junction sequence and enhanced stability. We found a substantial upregulation of circPIAS1 in HCC tissues, correlating with the TNM stage of HCC patients and reduced overall survival. Knockdown of circPIAS1 significantly induced ferroptosis and inhibited HCC cell proliferation and migration, both in vitro and *in vivo.* Mechanically, circPIAS1 acted as a sponge for miR-455-3p, upregulating its downstream target NUPR1. The circPIAS1/NUPR1 axis hindered ferroptosis activity in HCC by modulating FTH1 expression, exacerbating HCC progression (Fig. 9).

CircRNAs influence cancer evolution through various pathways [26]. Those containing microRNA response elements (MREs) can modulate miRNA-targeted mRNA expression by acting as miRNA sponges [27]. CircRNAs’ role as miRNA sponges in influencing miRNA-mediated gene expression in cancers is well-documented [28]. Additionally, circRNAs can employ other mechanisms. For example, circEIF3I may act as a molecular scaffold, interacting with SMAD3 and AP2A1 to form a complex that facilitates SMAD3 transport to early endosomes, activating downstream TGF-β signaling and promoting pancreatic cancer progression [29]. Some circRNAs can even encode proteins. For instance, in glioblastoma, circular E-cadherin RNA (circ-E-Cad) produces a secretory E-cadherin protein variant through multiple-round open reading frame (ORF) translation, activating EGFR signaling independent of EGF and maintaining glioma stem cell tumorigenicity [30]. We conducted bioinformatics analyses to determine whether circPIAS1 interacts with RNA-binding proteins or contains potential ORFs or ribosome binding sites. While predictive binding sites for common RNA binding proteins, such as HuR or EIF4A3, were found, no functional ORF was identified, suggesting that circPIAS1 may function through protein interaction, requiring further investigation. This study conclusively validates circPIAS1’s role as a miR-455-3p sponge in HCC cells. Given miRNAs’ multiple downstream targets, miR-455-3p likely regulates HCC progression through other targets as well.

Ferroptosis has emerged as an anticancer mechanism in various malignant tumors [31]. NUPR1 plays a crucial role in conferring resistance to ferroptosis in cancer cells [32]. Recent research highlights NUPR1 as a stress protein that prevents ferroptosis and regulates iron metabolism by transcriptionally activating lipocalin 2 (LCN2) [19]. Consistent with this, our study demonstrates that circPIAS1-mediated upregulation of NUPR1 renders HCC cells resistant to ferroptosis by transcriptionally activating FTH1, the heavy subunit of ferritin, which plays a crucial role in maintaining cellular iron balance. Excess iron amplifies cellular ROS through the Fenton reaction, exacerbating oxidative damage and closely regulating carcinogenesis [33]. FTH1 inhibits the accumulation of intracellular Fe^2+^ and reduces cancer cell sensitivity to ferroptosis. This suggests that NUPR1 may be a central regulator of iron metabolism in cancer cells. However, further investigation is needed to identify other iron-related targets downstream of NUPR1.

Drug resistance poses a significant challenge in cancer treatment [34]. HCC is highly resistant to drugs, with surgery being the primary treatment option for early-stage patients. Targeted therapies have limited efficacy [35, 36], with sorafenib, the first FDA-approved molecularly targeted drug, often leading to resistance within 6 months [37]. Lenvatinib, another first-line drug for advanced HCC, also faces resistance challenges, with over 60% of patients becoming resistant within a year [38]. Despite efforts to develop combination therapies, overall outcomes remain unsatisfactory. Our study shows that high circPIAS1 expression enhances HCC cell resistance to lenvatinib. The tissue-specific and stage-specific expression of circPIAS1 makes it a suitable therapeutic target candidate for HCC, offering a potential strategy to overcome lenvatinib resistance. Additionally, the link between ferroptosis and tumor resistance has garnered attention. Compounds like Erastin and GPX4 inhibitors induce ferroptosis, enhancing anticancer efficacy [39]. ZZW-115, a potent NUPR1 inhibitor, induces ROS accumulation and ferroptotic cell death [17], making it a promising candidate for HCC treatment. Studies have shown that ZZW-115 enhances cancer cell sensitivity to genotoxic agents by obstructing NUPR1’s nuclear translocation, decreasing the SUMOylation-dependent functions of DNA damage proteins [40]. Our study indicates that the combined use of lenvatinib and ZZW-115 significantly reduces HCC tumors through enhanced ferroptosis, suggesting a novel strategy for future cancer treatment.

## Conclusions

In summary, our study robustly establishes circPIAS1 as an oncogene in HCC progression. We have demonstrated its role in promoting HCC cell proliferation and migration while inhibiting ferroptosis. This study enhances our understanding of circRNA functions in HCC development and progression, suggesting that targeting the circPIAS1/miR-455-3p/NUPR1/FTH1 axis could be a promising therapeutic strategy for HCC.

### Electronic supplementary material

Below is the link to the electronic supplementary material.

Supplementary Material 1

Supplementary Material 2

Supplementary Material 3

Supplementary Material 4

Supplementary Material 5

Supplementary Material 6

## Data Availability

All data supporting the findings of this study are available from the corresponding authors upon reasonable request. Correspondence and requests for materials should be addressed to Yi-Fan Lian.
