# Supplementary figures and images for: CircPIAS1 promotes hepatocellular carcinoma progression by inhibiting ferroptosis via the miR-455-3p/NUPR1/FTH1 axis
Source: Mol Cancer. 2024 May 28;23:113. doi: 10.1186/s12943-024-02030-x (PMC11131253; doi:10.1186/s12943-024-02030-x)

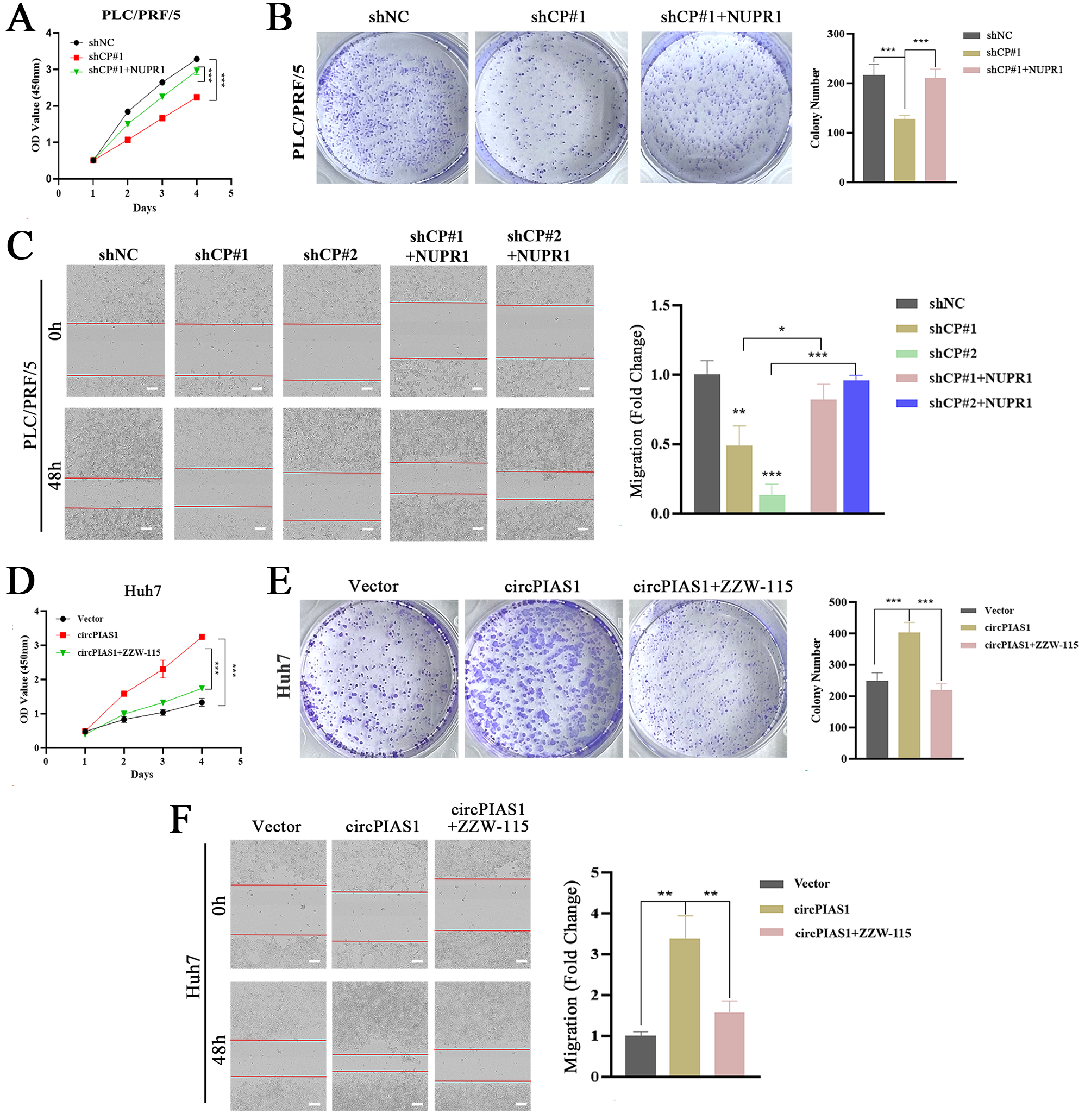

Supplement: Supplementary file 1 — Supplementary Material 1 [file 12943_2024_2030_MOESM1_ESM.tif]

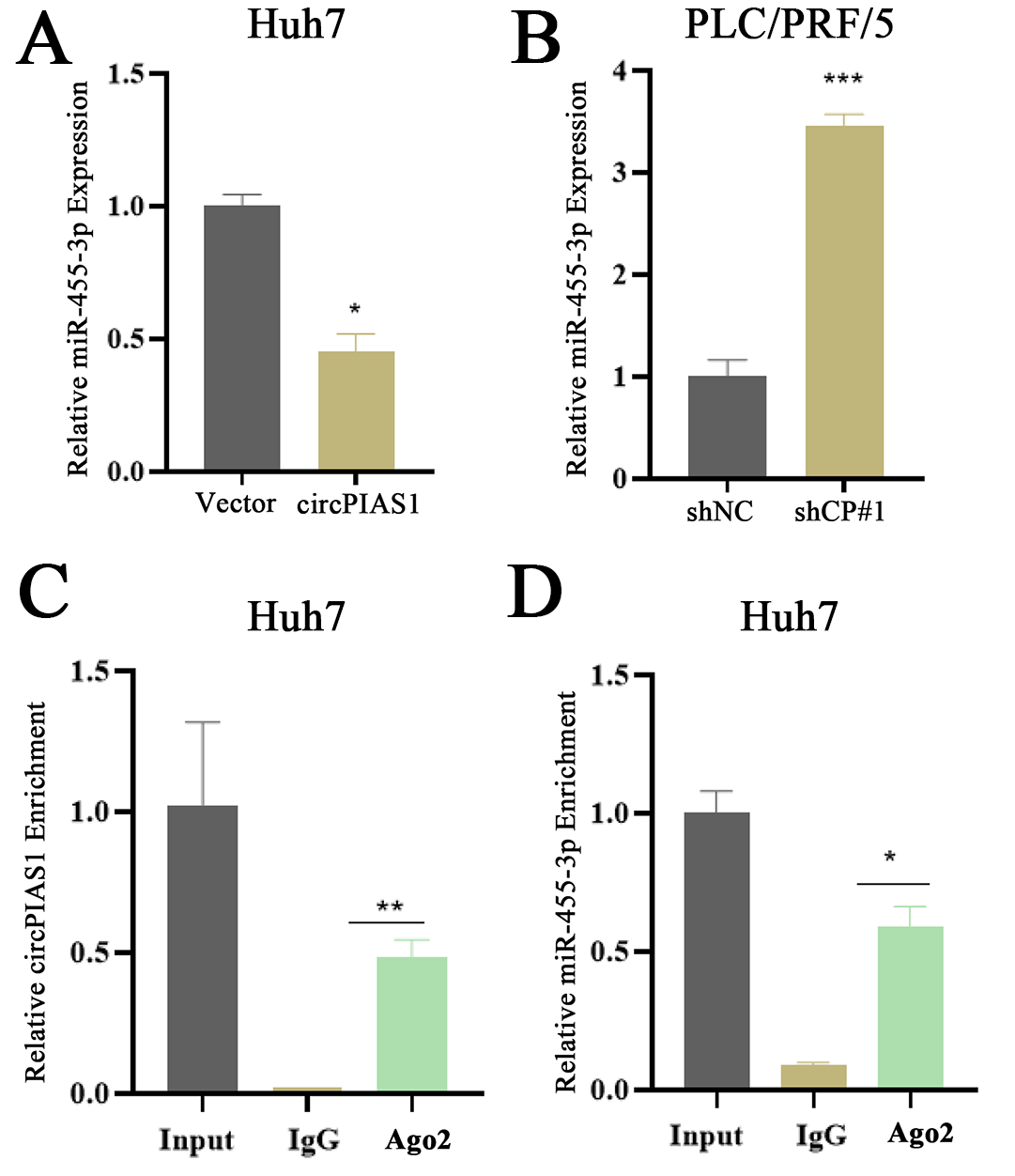

Supplement: Supplementary file 2 — Supplementary Material 2 [file 12943_2024_2030_MOESM2_ESM.tif]

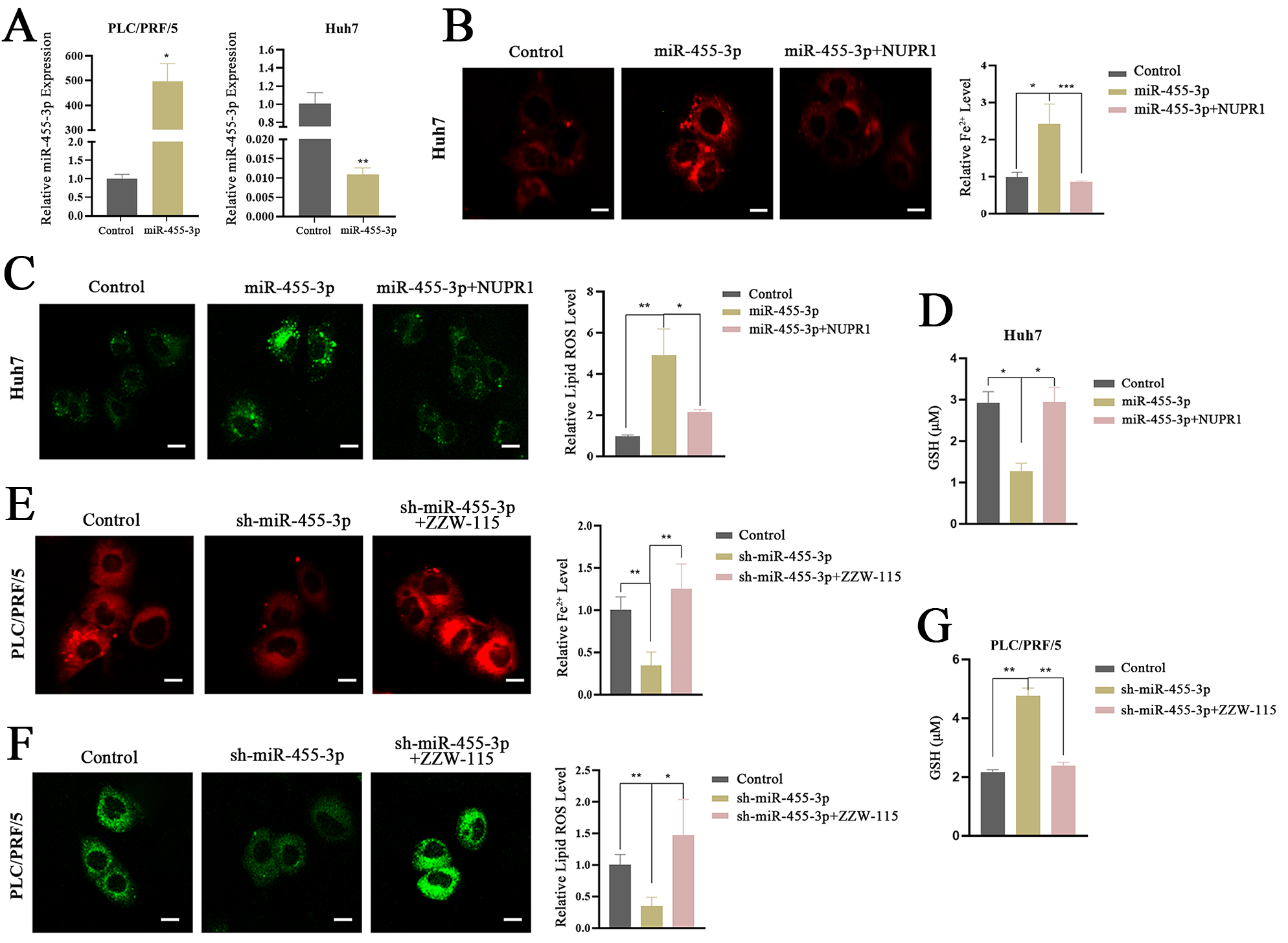

Supplement: Supplementary file 3 — Supplementary Material 3 [file 12943_2024_2030_MOESM3_ESM.tif]

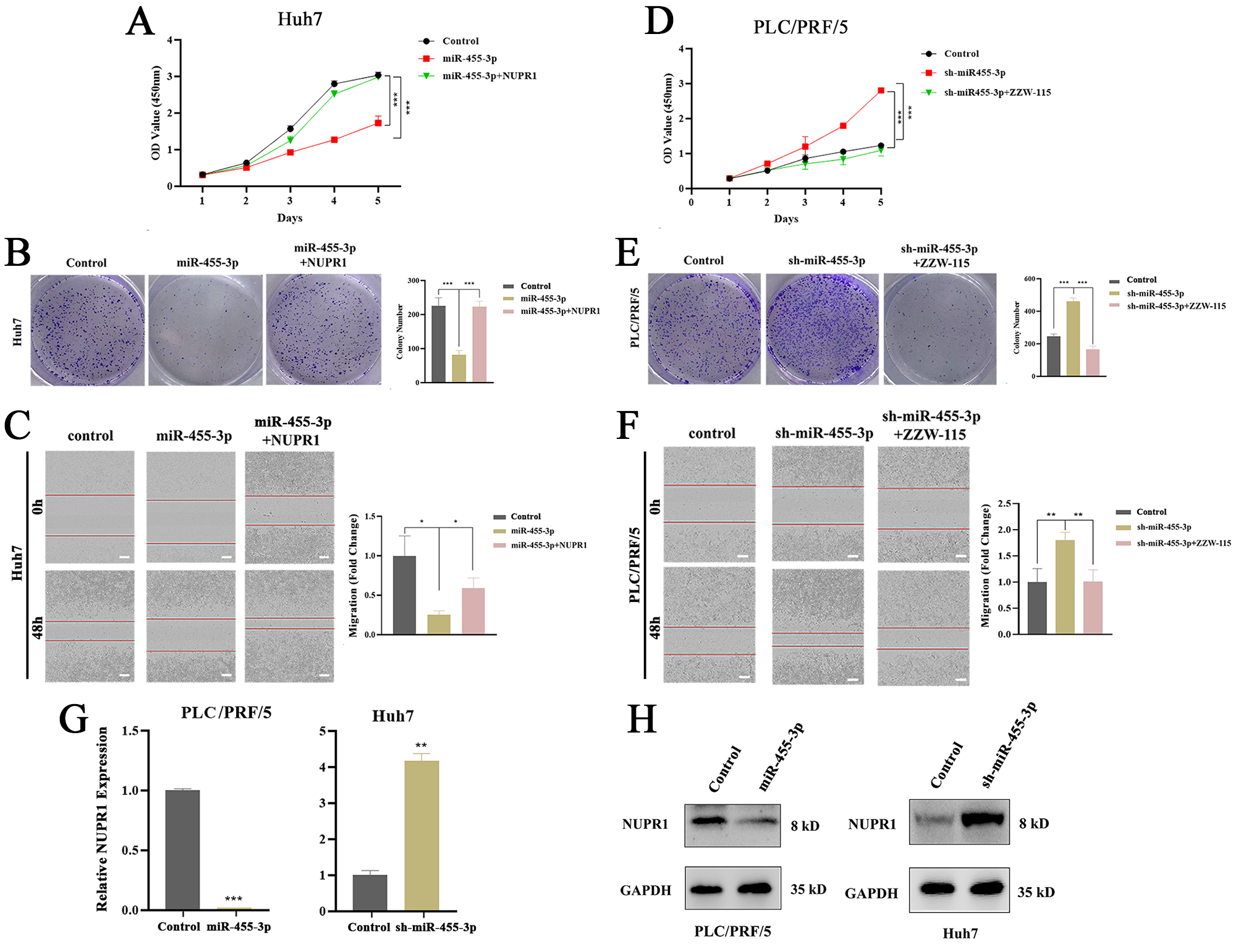

Supplement: Supplementary file 4 — Supplementary Material 4 [file 12943_2024_2030_MOESM4_ESM.tif]

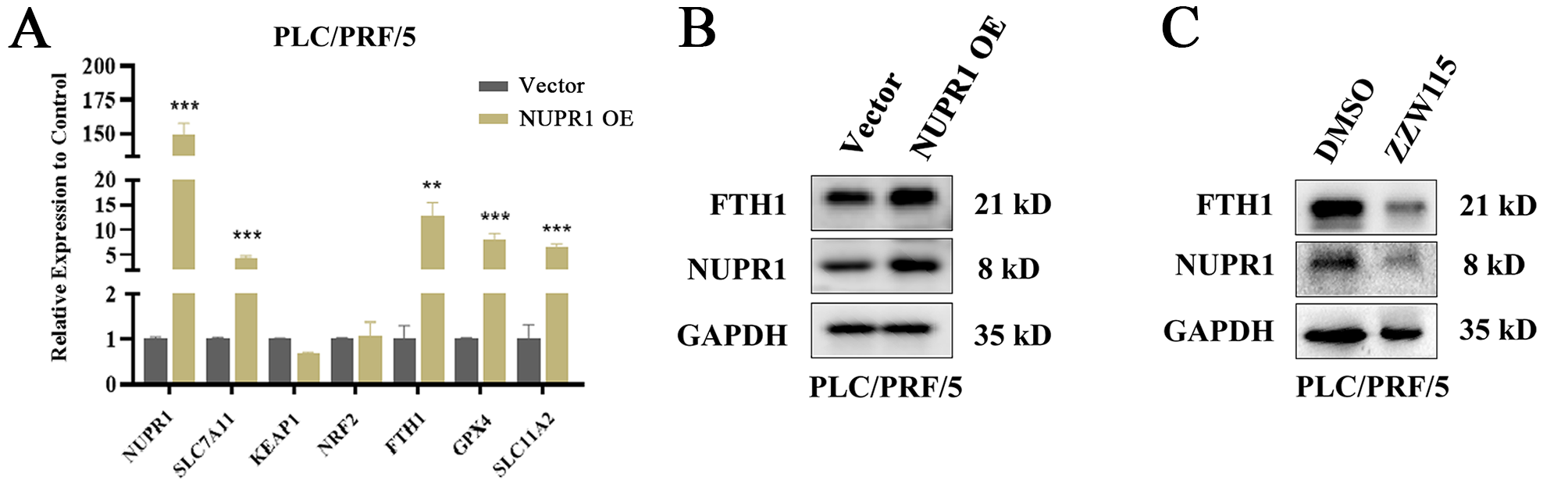

Supplement: Supplementary file 5 — Supplementary Material 5 [file 12943_2024_2030_MOESM5_ESM.tif]
