# Supplementary material for: CircPIAS1 promotes hepatocellular carcinoma progression by inhibiting ferroptosis via the miR-455-3p/NUPR1/FTH1 axis
Source: Mol Cancer. 2024 May 28;23:113. doi: 10.1186/s12943-024-02030-x (PMC11131253; doi:10.1186/s12943-024-02030-x)
Supplement: Supplementary file 6 — Supplementary Material 6 [file 12943_2024_2030_MOESM6_ESM.docx]

**Supplementary Table 1. Sequences of primers, probes and miRNA mimics or inihbitors.**

| **Names** | **Application** | **Sequences (5’→3’)** |
| --- | --- | --- |
| ***Primers*** |  |  |
| PIAS1 Forward | qPCR | ATTTGCCTTGACACCACAACA |
| PIAS1 Reverse | qPCR | CCTTAACTGGACCTGTACTGTGA |
| circPIAS1 Forward | qPCR | TCGGGCCCTTACATGTTCTC |
| circPIAS1 Reverse | qPCR | TGTTGTCTGATGCCATCAAT |
| FTH1 Forward | qPCR | CCCCCATTTGTGTGACTTCAT |
| FTH1 Reverse | qPCR | GCCCGAGGCTTAGCTTTCATT |
| GPX4 Forward | qPCR | GAGGCAAGACCGAAGTAAACTAC |
| GPX4 Reverse | qPCR | CCGAACTGGTTACACGGGAA |
| SLC7A11 Forward | qPCR | TCTCCAAAGGAGGTTACCTGC |
| SLC7A11 Reverse | qPCR | AGACTCCCCTCAGTAAAGTGAC |
| SLC11A2 Forward | qPCR | TGGAGATCATGGGGAGTCTG |
| SLC11A2 Reverse | qPCR | AAGAAAACCTGGTCCGGTGAA |
| KEAP1 Forward | qPCR | CTGGAGGATCATACCAAGCAGG |
| KEAP1 Reverse | qPCR | GGATACCCTCAATGGACACCAC |
| NRF2 Forward | qPCR | TCAGCGACGGAAAGAGTATGA |
| NRF2 Reverse | qPCR | CCACTGGTTTCTGACTGGATGT |
| NUPR1 Forward | qPCR | ACCTTCCCACCAGCAACC |
| NUPR1 Reverse | qPCR | ACCTTTCCGGCCTCCACCTC |
| GAPDH Forward | qPCR | GAAGGTGAAGGTCGGAGTC |
| GAPDH Reverse | qPCR | GAAGATGGTGATGGGATTTC |
| U6 Forward | qPCR | CGCTTCGGCAGCACATATACTA |
| U6 Reverse | qPCR | CGCTTCACGAATTTGCGTGTCA |
| FTH1 promoter primer1 Forward | ChIP-qPCR | AGGTGTCTCTATGCTGTCT |
| FTH1 promoter primer1 Reverse | ChIP-qPCR | GCAGGAGAATCGCTTGAA |
| FTH1 promoter primer2 Forward | ChIP-qPCR | CCAGGTTCAAGCGATTCT |
| FTH1 promoter primer2 Reverse | ChIP-qPCR | TGTGGAAATGTGGAAATGC |
| FTH1 promoter primer3 Forward | ChIP-qPCR | CTGTTCACTCTGCTCCAA |
| FTH1 promoter primer3 Reverse | ChIP-qPCR | GAAGGATGCTGCTAGACC |
| FTH1 promoter primer4 Forward | ChIP-qPCR | CCTAATTCCGTCGGCAAT |
| FTH1 promoter primer4 Reverse | ChIP-qPCR | GAGGTCCACTGTTCTGTC |
| FTH1 promoter primer5 Forward | ChIP-qPCR | TGCCACCCATAAATTCACA |
| FTH1 promoter primer5 Reverse | ChIP-qPCR | CACTCACAGGAGCTTCAG |
| FTH1 promoter primer6 Forward | ChIP-qPCR | CGACACCAGCGATTCTCT |
| FTH1 promoter primer6 Reverse | ChIP-qPCR | CTCAGAGTCCAGGAAGGAG |
| FTH1 promoter primer7 Forward | ChIP-qPCR | GTCTCCACCTTCCACCTT |
| FTH1 promoter primer7 Reverse | ChIP-qPCR | GAAGTCCACCTCAGAACC |
| circPIAS1 divergent Forward | RT-PCR | TCGGGCCCTTACATGTTCTC |
| circPIAS1 divergent Reverse | RT-PCR | CCTTAACTGGACCTGTACTG |
| circPIAS1 convergent Forward | RT-PCR | AAATGGCGTGGAACCAAAGC |
| circPIAS1 convergent Reverse | RT-PCR | TGGTACTGTTGTGGACAGTCG |
| GAPDH divergent Forward | RT-PCR | GAAGACTGTGGATGGCCCCT |
| GAPDH divergent Reverse | RT-PCR | CAAATGAGCCCCAGCCTTCT |
| GAPDH convergent Forward | RT-PCR | GGAGCGAGATCCCTCCAAAAT |
| GAPDH convergent Reverse | RT-PCR | GGCTGTTGTCATACTTCTCATGG |
| ***Probes*** |  |  |
| circPIAS1 Probe | RNA pulldown | UCUCGAAAGCGCUGACUGUUGUCUGAUGCCAUCAAUAAUAAGGUGUUCAUAUGGAGCCUUCUU |
| Control probe | RNA pulldown | UUGUACUACACAAAAGUACUG |
| hsa-miR-455-3p probe | FISH | AGTGTATATGCCCATGGACTGCA |
| circPIAS1 Probe | FISH | GACTGTTGTCTGATGCCATCAATAATAAGGT |
| ***miRNAs*** |  |  |
| hsa-miR-455-3p mimics | miRNA | GCAGUCCAUGGGCAUAUACAC |
| hsa-miR-455-3p inhibitors | miRNA | CGUCAGGUACCCGUAUAUGUG |
| mimics NC | miRNA | UUUGUACUACACAAAAGUACUG |
| inhibitors NC | miRNA | CAGUACUUUUGUGUAGUACAAA |
